# The Prefoldin Complex Regulates Chromatin Dynamics during Transcription Elongation

**DOI:** 10.1371/journal.pgen.1003776

**Published:** 2013-09-19

**Authors:** Gonzalo Millán-Zambrano, Alfonso Rodríguez-Gil, Xenia Peñate, Lola de Miguel-Jiménez, Macarena Morillo-Huesca, Nevan Krogan, Sebastián Chávez

**Affiliations:** 1 Departmento de Genética, Universidad de Sevilla, and Instituto de Biomedicina de Sevilla, Hospital Universitario Virgen del Rocío/CSIC/Universidad de Sevilla, Seville, Spain; 2 Department of Cellular and Molecular Pharmacology, University of California San Francisco, San Francisco, California, United States of America; Indiana University, Howard Hughes Medical Institute, United States of America

## Abstract

Transcriptional elongation requires the concerted action of several factors that allow RNA polymerase II to advance through chromatin in a highly processive manner. In order to identify novel elongation factors, we performed systematic yeast genetic screening based on the GLAM (Gene Length-dependent Accumulation of mRNA) assay, which is used to detect defects in the expression of long transcription units. Apart from well-known transcription elongation factors, we identified mutants in the prefoldin complex subunits, which were among those that caused the most dramatic phenotype. We found that prefoldin, so far involved in the cytoplasmic co-translational assembly of protein complexes, is also present in the nucleus and that a subset of its subunits are recruited to chromatin in a transcription-dependent manner. Prefoldin influences RNA polymerase II the elongation rate *in vivo* and plays an especially important role in the transcription elongation of long genes and those whose promoter regions contain a canonical TATA box. Finally, we found a specific functional link between prefoldin and histone dynamics after nucleosome remodeling, which is consistent with the extensive network of genetic interactions between this factor and the machinery regulating chromatin function. This study establishes the involvement of prefoldin in transcription elongation, and supports a role for this complex in cotranscriptional histone eviction.

## Author Summary

Transcription is the biological process that allows genes to be copied into RNA; the molecule that can be read by the cell in order to fabricate its structural components, proteins. Transcription is carried out by RNA polymerases, but these molecular machines need auxiliary factors to guide them through the genome and to help them during the RNA synthesis process. We searched for novel auxiliary factors using a genetic procedure and found a set of potential novel transcriptional players. Among them, we encountered a highly unexpected result: a factor, called prefoldin, so far exclusively involved in the folding of proteins during their fabrication. We confirmed that prefoldin binds transcribed genes and plays an important role during gene transcription. We also further investigated this transcriptional role and found that prefoldin is important for unpacking genes, thus facilitating the advance of the RNA polymerases along them.

## Introduction

RNA polymerase II transcribes nuclear protein-coding genes in eukaryotes. After the assembly of the preinitiation complex onto the promoter and the subsequent initiation of transcription, the enzymatic activity of RNA polymerase II enables the synthesis of considerably long RNA molecules in the elongation phase of transcription. During this process, nascent RNA is modified by capping and polyadenylation, exons are defined for splicing, while hnRNP components are recruited, thus ensuring the production of mature mRNAs that can be exported to the cytoplasm [1].

This complexity explains the tight regulation of transcription elongation, which involves a large number of auxiliary factors. Some of these factors contribute to transcription elongation by controlling the cotranscriptional processes affecting nascent RNA [2] or by modulating the processivity of RNA polymerase II by either favoring or preventing its stalling [3], [4]. When paused, RNA polymerase II can arrest after backtracking [5]. In this case, factors like TFIIS stimulate the RNA cleavage activity of RNA polymerase II to enable it to resume transcription elongation [6]–[8].

Since the physiological substrate of transcription is chromatin, transcription elongation is one of the main processes that contribute to shape the nucleosomal landscape of the genome [9] and the distribution of histone modification marks [10]. Accordingly, a large set of auxiliary factors are involved in facilitating transcription elongation through chromatin [11]. Nucleosome remodeling machines, like RSC, utilize ATP-dependent helicases to destabilize nucleosomes during transcription [12]. Other chromatin factors, like FACT, contribute to the disassembly and reassembly of nucleosomes during transcription elongation in an ATP-independent manner [13]. Other examples are the chromatin remodelers Isw1 and Chd1, which prevent histone exchange during transcription [14]. The action of these factors is often coupled to specific histone covalent modifications, which are carried out by a different set of auxiliary players; for example, the Rpd3S complex which de-acetylates histone tails [15], or Set2, which methylates the K36 residue of histone H3 [16], and thereby represses histone exchange [17].

One last group of elongation factors controls the phosphorylation state of RNA polymerase II, particularly the carboxyterminal domain of its largest subunit. This CTD domain is the docking platform that allows some of the other above-described elements to act during transcription elongation. Accordingly, the factors affecting CTD phosphorylation control the overall integration of the molecular functions required to complete the transcription cycle [18].

The number of protein complexes in the literature playing specific roles during transcription elongation has increased in the last few years, suggesting that our knowledge on this field is still partial. In order to detect novel factors involved in transcriptional elongation, we performed a systematic genetic screening of a collection of *Saccharomyces cerevisiae* viable deletions. Using an assay based on the comparison of transcription units whose transcribed region length differs, we detected novel genes that have not been previously linked to transcription elongation. Among them, we found *PFD1* encoding a subunit of the prefoldin complex.

Prefoldin acts as a co-factor of group II chaperonins in eukaryotes and archaea [19]. Prefoldin forms jellyfish-shaped hexameric complexes consisting of two alpha-type and four beta-type subunits [20]–[22]. It is functionally involved in the cotranslational folding of proteins like tubulin and actin by presenting unfolded polypeptides to cytosolic chaperonin CCT [23], [24]. All six prefoldin genes are non-essential in yeast, although their deletion is deleterious under conditions that stress microtubules and actin dynamics [25], [26]. The mutation of prefoldin genes causes embryonic lethality in *Caenorhabditis elegans* and produces severe cytoskeletal defects in mice that promote their early death [27]. Nuclear functions of prefoldin are scarce in the literature and it has not been previously connected to transcription elongation. In this work we describe our finding of the transcriptional function of prefoldin by showing that it is present in the nucleus, binds chromatin in a transcription-dependent manner and contributes to chromatin dynamics during transcription elongation.

## Results

### Genetic screening based on the GLAM assay uncovers novel potential factors involved in transcriptional elongation

**Figure 1:**
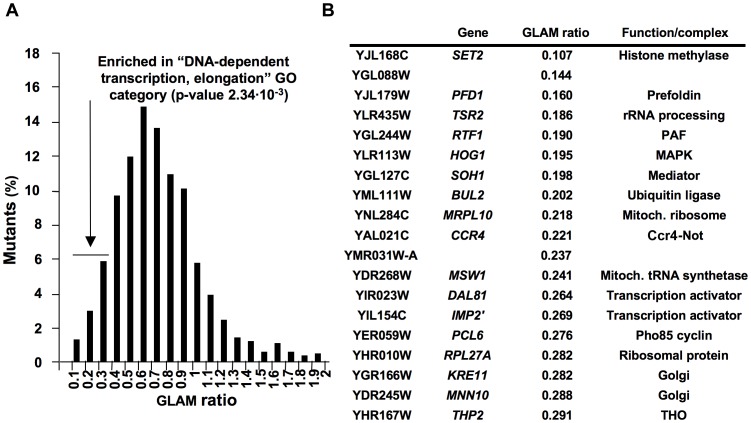
Systematic genetic analysis of budding yeast *Saccharomyces cerevisiae* based on the GLAM assay. GLAM ratios were calculated for the mutants present in the collection of viable deletions, growing in microtiter plates, as described in Materials and methods. The distribution of the values obtained is represented in **A**. The deletions exhibiting GLAM ratios lower than 0.3 were significantly enriched in the indicated gene ontology category. Those low-scored mutants that maintained low GLAM ratios in exponentially growing cells are listed in **B**.

The GLAM (gene length-dependent accumulation of mRNA) assay is a useful tool to detect defects in mRNA biogenesis that occur in a gene length-dependent manner. By comparing two transcription units, both encoding the acid phosphatase Pho5 and whose only difference lies in the length of their 3′ UTR, we can easily detect gene mutations that specifically decrease the expression level of the long unit without affecting the short one [28]. We previously made full use of the GLAM assay to establish functional links between already known transcription complexes, like SAGA, the Mediator or the Ccr4-Not complex, and transcription elongation [29]. In order to identify novel elements that play a role in transcription elongation, we carried out a systematic genetic screening by performing GLAM assays using the collection of viable deletion mutants in *Saccharomyces cerevisiae*. To do this, we introduced plasmid pGAL1-PHO5 and pGAL1-PHO5::lacZ (Figure S1A) into all the deleted strains by crossing, sporulating and selecting appropriate markers (Figure S1B). After cultivating all the transformants in microtiter plates, acid phosphatase activity was measured (Figure S1B). In all, 1878 mutants produced reproducible and significant levels of activity with the two plasmids. GLAM ratios were calculated by dividing the phosphatase activity of the cells expressing the long unit by the activity of the cells expressing the short one (Table S1). Values ranged from 0.1 to 1.9 and they shaped a Gaussian distribution that peaked between 0.6 and 0.7, which was slightly below the 0.8 units of the wild-type GLAM ratio (Figure 1A). No specific gene-ontology category was significantly overrepresented among those mutants showing high GLAM ratios, but the mutants scoring between 0.1 and 0.3 were seen to be significantly enriched in “DNA-dependent transcription, elongation” (p value 2.34 ·10^−3^) and in “gene expression” (p value 3.08 · 10^−3^) (Figure 1A). We repeated the GLAM assays of all these mutants after cultivating them under conditions that ensured exponential growth. Those that were confirmed in this analysis are listed in Figure 1B. The lowest score on the screen was observed with a strain deleted for *SET2*, which codes for a histone H3 methytransferase, an enzyme that has been clearly connected to transcription elongation by RNA polymerase II [30], [31]. Similarly, other mutants in this list, like *rtf1Δ* (PAF complex), *soh1Δ* (Mediator), *ccr4Δ* (Ccr4-Not complex) and *thp2Δ* (THO complex), lacked general transcriptional machinery elements that are directly or indirectly involved in transcription elongation [32]–[35]. A number of genes encoding transcription activators (Dal81, Imp2′) [36], [37] or proteins functionally linked to RNA polymerase II (Hog1) [38] were also found. Although the GLAM assay is able to detect any mutant affecting gene expression in a length-dependent manner, mutants related to posttranscriptional aspects, like mRNA stability or translation, were not found. Finally, several mutants with very low scores have never been related to transcription elongation, or even to gene expression (Figure 1B). This study establishes a preliminary link between these genes and mRNA biogenesis, which is to be confirmed by additional studies.

### Prefoldin mutants exhibit transcriptional defects

**Figure 2:**
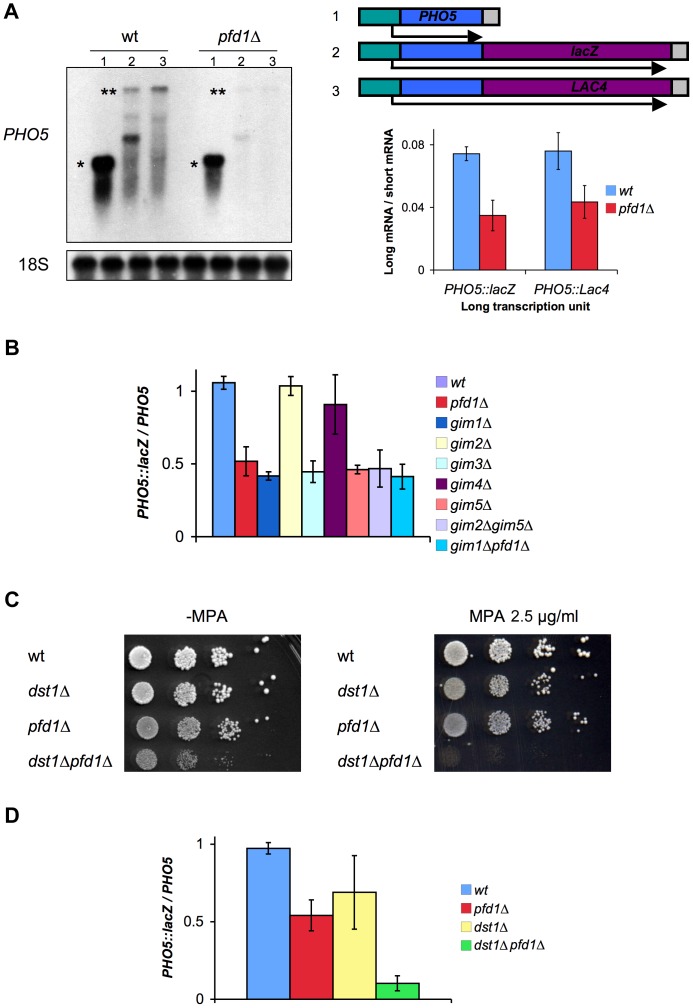
Prefoldin mutants exhibit transcriptional phenotypes and synthetic interactions with TFIIS. **A.** *pfd1Δ* was impaired in the accumulation of 4.5 kb long mRNAs (**), but behaved as the wild type when expressing a 1.5 kb mRNA (*) driven by the same promoter. A single short transcription units (1) was utilized, but two different long transcription units were tested in order to exclude sequence-specific defects: *GAL1pr-PHO5::lacZ* (2) and *GAL1pr-PHO5::LAC4* (3). A significant Northern experiment is shown on the left. The average ratios calculated dividing the signals of the long transcript by the signal of the short transcript are shown on the right. Values correspond to the mean and the standard deviation of three biological replicates. **B.** Four out of six prefoldin mutants, lacking the yeast prefoldin complex subunits, exhibited significantly lower GLAM ratios than the wild type. The GLAM ratios of double mutants were no lower than single ones. GLAM ratios were calculated dividing the acid phosphatase activity of cells expressing *GAL1pr-PHO5::lacZ* by the activity of cells of the same strain expressing *GAL1pr-PHO5*. Values correspond to the mean and the standard deviation of three biological replicates. **C.** The *pfd1Δ dst1Δ* double mutant exhibited hypersensitivity to 2.5 µg/ml mycophenolyc acid. Ten-fold serial dilutions were used for the drop assay. **D.** The *pfd1Δ dst1Δ* double mutant showed synergistically lower GLAM ratios, as compared to the corresponding single mutants. The GLAM ratios in B and D were calculated after measuring the acid phosphatase activity encoded in the GLAM transcription units, and were normalized to the wild-type value to facilitate comparisons. Values correspond to the mean and the standard deviation of three biological replicates.

We decided to focus on *PFD1*, which provided the third most significant effect in the GLAM assay when deleted (Figure 1B). We confirmed the GLAM phenotype of *pfd1Δ* by Northern blot. The levels of the full-length mRNAs transcribed from *GAL1p-PHO5::lacZ* were significantly reduced in *pfd1Δ* as compared to the short mRNA from *GAL1p-PHO5* (Figure 2A). The same results were obtained for a second construct, in which the 3′UTR sequence was different (*GAL1p-PHO5::LAC4*) [28], indicating that the effect is not sequence specific (Figure 2A).

*PFD1* encodes one of the components of prefoldin, a heterohexameric complex composed of two alpha subunits (Gim2 and Gim5) and four beta subunits (Gim1, Gim3, Gim4 and Pfd1) [39]. We tested the GLAM phenotypes of the mutants lacking the other five subunits. We found that *gim1Δ*, *gim3Δ* and *gim5Δ* also gave GLAM ratios that were significantly below the wild type (Figure 2B). We also analyzed some double mutants. The *gim2Δ gim5Δ* mutant, lacking the two alpha subunits, gave the same GLAM values as the single *gim5Δ* mutant (Figure 2B), indicating that only one alpha subunit type is involved in the transcriptional function of prefoldin. We also combined two deletions that exhibit the GLAM phenotype: *gim1Δ* and *pfd1Δ*. The resulting double mutant did not give significantly lower GLAM values than the corresponding single mutants (Figure 2B). We conclude that the role of these beta subunits is not redundant and that they cannot substitute each other in the transcriptional function of the complex.

Since prefoldin is involved in the cytoplasmic folding of microtubules and actin filaments, we explored the possibility of prefoldin GLAM phenotypes resulting from cytoskeleton defects. To test this, before the assay we treated the cells with benomyl, a powerful inhibitor of microtubules dynamics. We found neither a significant effect of this drug on the GLAM ratios in the wild type (Figure S2A) nor a direct correlation in prefoldin mutants between their sensitivity to benomyl and their GLAM phenotype (Figure S2B). Moreover, *pfd1Δ*, with a low GLAM ratio, showed very mild sensitivity to benomyl (Figure S2B). Similarly, prefoldin mutants exhibited uneven sensitivity to latrunculin A, an inhibitor of actin polymerization (Figure S2C). Whereas *pfd1Δ* was no more sensitive than the wild type, *gim2Δ*, which displayed identical GLAM ratios to the wild type, clearly showed hypersensitivity to this drug. These results support a functional contribution of some prefoldin subunits to gene expression which, at least in the case of Pfd1, might be even more relevant than their role in cytoskeleton folding.

The GLAM phenotype of prefoldin mutants suggests the involvement of prefoldin in transcription elongation. The only known connection between prefoldin and transcription elongation derives from genetics since it has been described that *gim1Δ*, *gim3Δ* and *gim5Δ* display a synthetic growth defective phenotype when combined with *dst1Δ*, the mutant lacking RNA cleavage factor TFIIS [40]. We confirmed this synthetic growth phenotype and extended it to *pfd1Δ* (Figure 2C). Some transcription elongation mutants, such as *dst1Δ*, are sensitive to NTP-depleting drugs [41]. It is noteworthy that, although none of the prefoldin mutants was sensitive to high mycophenolyc acid concentrations (not shown), the *pfd1Δ dst1Δ* double mutant exhibited significantly enhanced sensitivity to very low concentrations of this drug (Figure 2C), and of 6-azauracil (not shown). This synthetic genetic interaction between *PFD1* and *DST1* was also detected by performing the GLAM assay, as the double *pfd1Δ dst1Δ* mutant exhibited synergistically lower GLAM ratios (Figure 2D). Altogether, these results support that the synthetic interaction between *PFD1* and *DST1* takes place in the transcription elongation context.

### Prefoldin subunits localize to the nucleus and a subset of them are recruited to chromatin in a transcription-dependent manner

**Figure 3:**
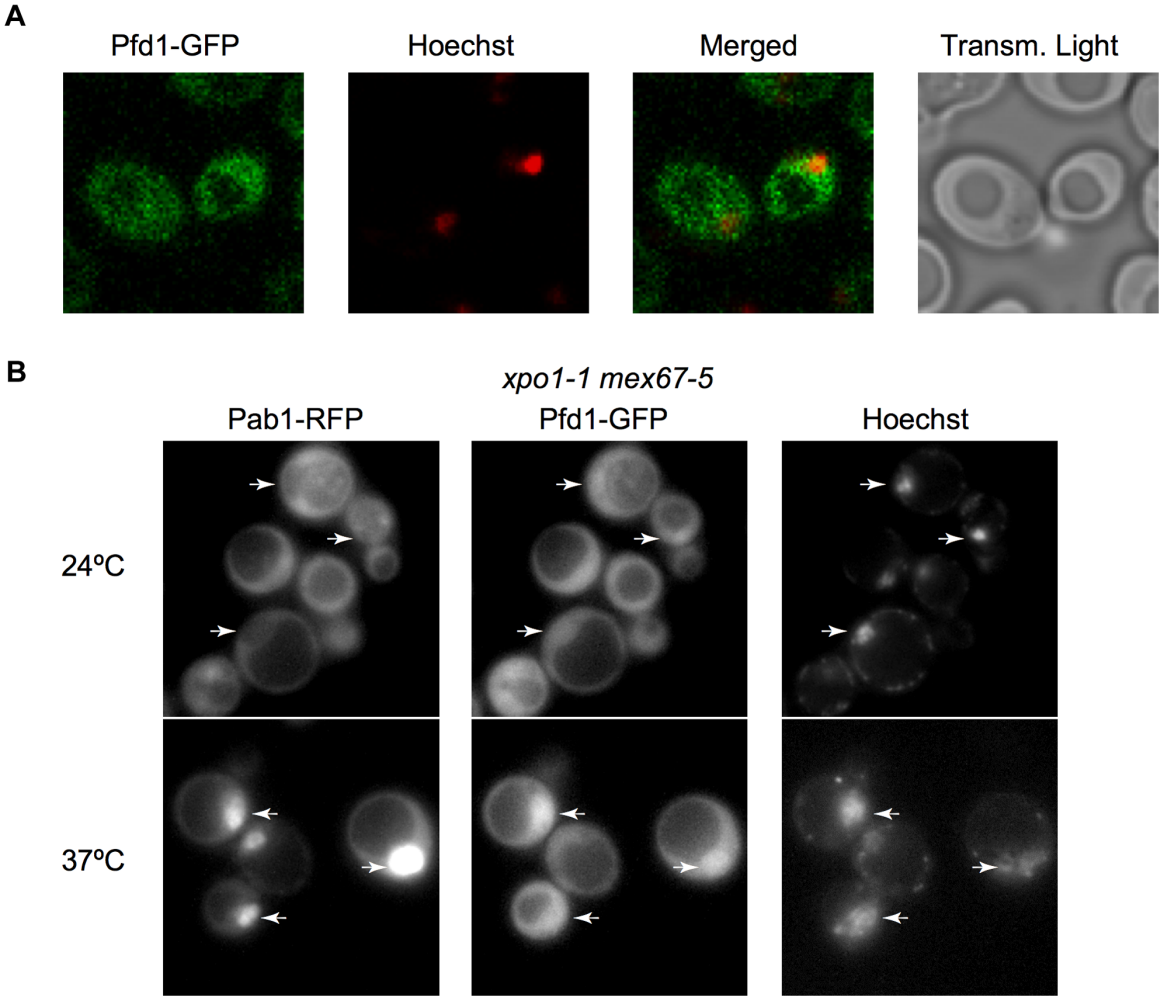
Prefoldin localizes to the nucleus. **A.** Analysis of cells expressing Pfd1-GFP by confocal microscopy indicates a nucleo-cytoplasmic distribution of this protein. Confocal microscopy was performed as described in the Materials and methods section. Nuclei were stained with Hoechst. **B.** Nucleo-cytoplasmic distribution of Pfd1-GFP shifts to nuclear upon inactivation of exportins. Cells of the thermosensitive *xpo1-1 mex67-5* double mutant expressing Pfd1-GFP were grown at 24°C and then incubated at 37°C during 4 h. Cells under these two conditions were observed using a fluorescence microscope, as described in the Materials and methods section. An RFP fusion of the well-known Pab1 shuttling protein is shown as a control in the same cells [42]. Arrows indicate nuclei, which were stained with Hoechst. Functionality of Pfd1-GFP is shown in Figure S3A.

Although it was first described as a cytoplasmic complex [25], a direct role of prefoldin in transcription would involve its nuclear localization. . We constructed a Pfd1-GFP fusion (Figure S3A) and found that it exhibits nucleo-cytoplasmic localization (Figure 3A). Does Pfd1 need active transport to enter and leave the nucleus? To test this, we used the export thermosensitive mutant *xpo1-1 mex67-5*. In this mutant, Pab1-RFP (used here as a control of the assay) is excluded from the nucleus at the permissive temperature and accumulates in the nucleus at the restrictive temperature [42](Figure 3B). As expected from the result in Figure 3A, Pfd1-GFP was not excluded from the nucleus at the permissive temperature, but accumulated at the restrictive temperature, indicating that prefoldin is a substrate of the active mechanisms of nucleo-cytoplamic export (Figure 3B). Similar results were obtained with Gim5-GFP (not shown). The other prefoldin subunits also exhibited nucleo-cytoplasmic localization (Figure S3B). We conclude that the presence of prefoldin subunits in the nucleus is consistent with a role of this complex during transcription elongation.

**Figure 4:**
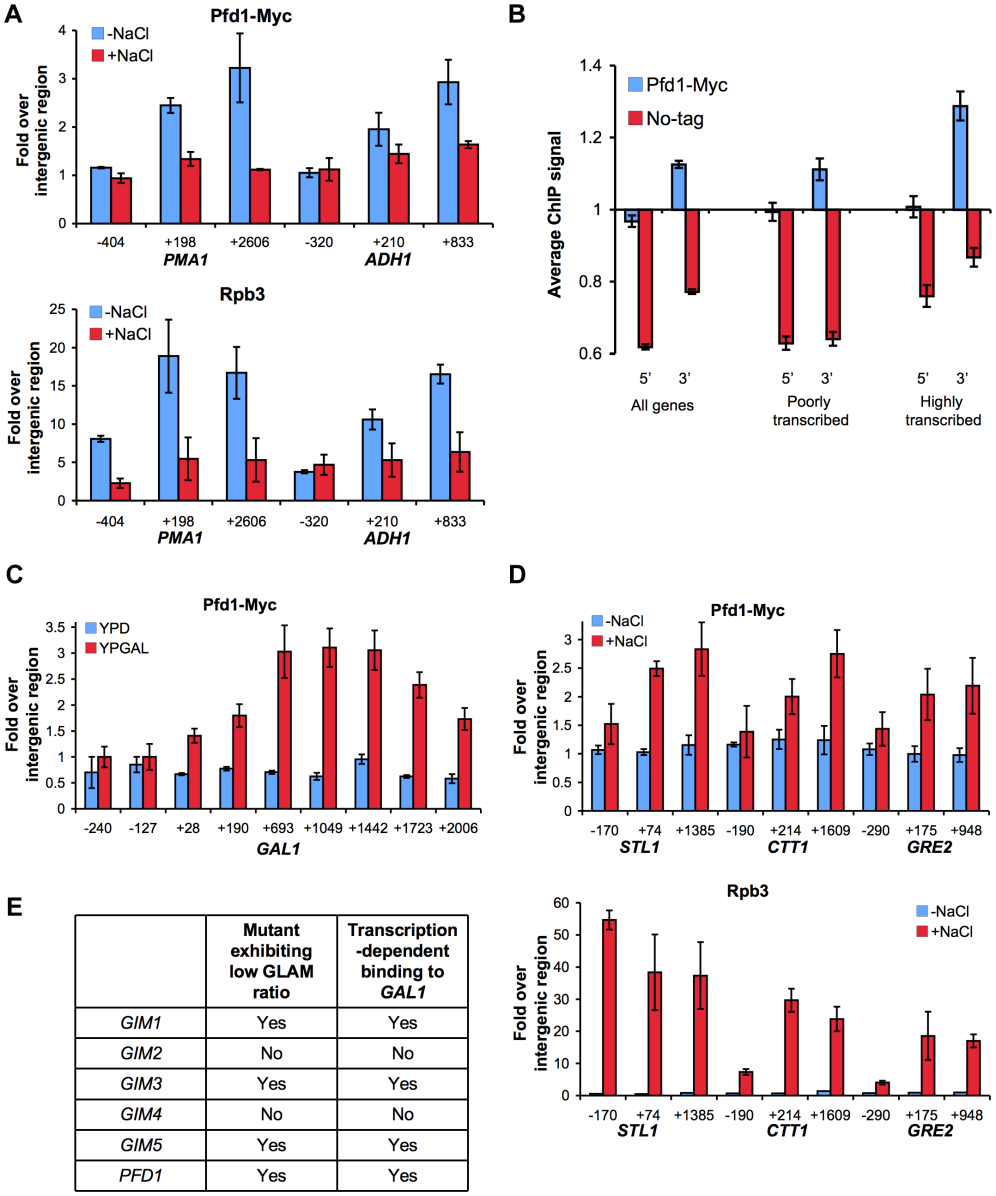
Prefoldin binds the transcribed region of active genes. **A.** Pfd1 binds the transcribed regions of *ADH1* and *PMA1* in a transcription dependent manner. Binding of Pfd1-Myc and Rpb3 to the indicated regions of these two genes was analyzed in cells exponentially growing in YPD medium, before and 10 min after adding 0.4 M NaCl. Pfd1-Myc and Rpb3 occupancy was analyzed by ChIP, as described in the Materials and methods section. Values correspond to the mean and the standard deviation of three biological replicates. **B.** Pfd1-Myc preferentially binds the 3′ ends of transcribed yeast genes. Pfd1-Myc occupancy across the genome was analyzed by ChIP on chip using Affymetrix tiling arrays, as described in Materials and methods. Average signal of 5′ and 3′ regions (300 bp long) were calculated for all protein-coding genes, and for the 10% showing the highest and the lowest Rpb3 occupancy, according to parallel Anti-Rpb3 ChIP on chip analysis. Mean values and standard error are shown for isogenic Pfd1-Myc and no-tag strains. The linear scale was optimized to visualize the difference with no-tag controls. Significance of this difference, according to the Mann-Whitney test, is commented in the main text. **C.** Pfd1 binds the transcribed region of *GAL1* under galactose-dependent activation conditions. Pfd1-Myc cells exponentially growing in glucose- (YPD) or in galactose-containing medium (YPGAL) were analyzed by ChIP, as described in the Materials and methods section. Values correspond to the mean and the standard deviation of three biological replicates. **D.** Pfd1 binds the transcribed region of *STL1*, *CTT1* and *GRE2* in response to osmotic stress. Binding of Pfd1 and Rpb3 to the indicated regions of these two genes were analyzed in cells exponentially growing in YPD medium, before and 10 min after adding NaCl up to 0.4 M. Pfd1-Myc and Rpb3 occupancy was analyzed by ChIP, as described in the Materials and methods section. Values correspond to the mean and the standard deviation of three biological replicates. **E.** Correlation between the GLAM phenotype of the prefoldin mutants and the binding of the corresponding proteins to *GAL1* in a transcription-dependent manner, according to the data shown in Figures 2B, 4C and S4B–C.

To address whether prefoldin directly regulates transcription by binding to chromatin we constructed a Pfd1-Myc fusion (Figure S4A) and carried out chromatin immunoprecipitation (ChIP) experiments using anti-Myc antibodies. The obtained results revealed that prefoldin is physically associated with the coding region of actively transcribed genes like *ADH1* and *PMA1* (Figure 4A, blue bars). The detected level of Pfd1-Myc association with the transcribed region was clearly lower than RNA polymerase II, but was comparable or even higher than well-known elongation factors like TFIIS [43], or chromatin factors that act during transcription elongation like SAGA [44],

Occupancy was stronger in the 3′ end of the genes tested (Figure 4A). In order to check if this was a general feature of prefoldin binding, we investigated Pfd1-Myc binding genome-wide. Although the overall signal was low, we detected a certain degree of binding of the 3′ gene ends by Pfd1-Myc (Figure 4B). No gene ontology categories were significantly enriched in those genes exhibiting higher Pfd1-Myc occupancy. However, average binding at 3′ was stronger for the 10% genes exhibiting the highest RNA polymerase II occupancy than for the least transcribed decile (Figure 4B). Accordingly the difference between Pfd1 binding in 5′ and 3′ was more significant in highly transcribed genes (p-value 1.1·10^−10^ after Mann-Whitney test) than in genes exhibiting poor RNA polymerase II occupancy (p-value 3.9· 10^−3^).

The results above suggest that prefoldin recruitment is influenced by transcriptional activity. To confirm this, we analyzed Pfd1 binding to inducible genes like *GAL1*, under active and inactive conditions. We found that Pfd1 binding to *GAL1* was transcription-dependent since the ChIP signal in the coding region was found exclusively under culture conditions where *GAL1* transcription was active (galactose-containing medium) and not under repressive conditions (Figure 4C). Similar results were found for Gim1, Gim3 and Gim5, but no significant binding of Gim2 and Gim4 to *GAL1* was detected (Figures S4B–C). We also tested the recruitment of Pfd1 to three osmotic stress-responsive genes (*STL1*, *CTT1* and *GRE2*) that become transiently induced upon salt treatment [45]. As shown in Figure 4D, Pfd1 did not show any occupancy of these genes under non-stress conditions, whereas Pfd1 was recruited to their coding region only 10 min after the addition of salt and in parallel to RNA polymerase II (Figure 4D). In all the genes tested, Pfd1 occupancy was greater at the 3′ end than at 5′ (Figures 4A, C, D).

It has been previously shown that some housekeeping genes undergo strong reduction of RNA polymerase II occupancy in response to osmotic stress [45]. We found that 10 min after salt addition, and again in parallel to RNA polymerase II occupancy, Pfd1 binding to *PMA1* and *ADH1* diminished (Figure 4A). Altogether, these results support that prefoldin recruitment to chromatin depends on transcription.

**Figure 5:**
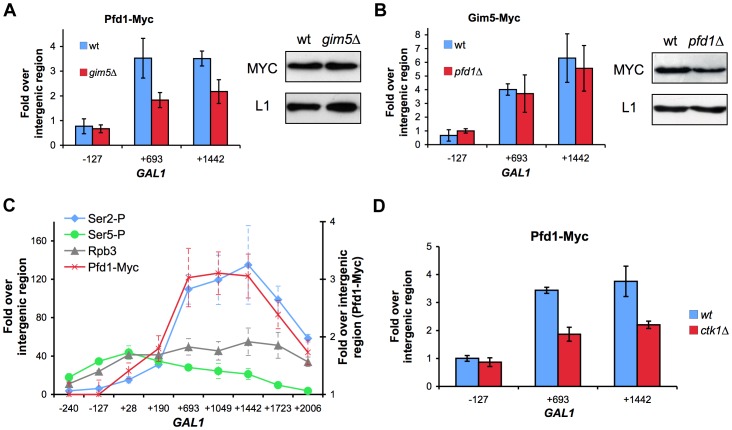
Recruitment of prefoldin to transcribed genes depends on the integrity of the complex and is favored by Ser2-phosphorylation of the Rpb1 CTD. **A.** Pfd1 binding to *GAL1* partially depends on Gim5. Pfd1-Myc occupancy of *GAL1* was analyzed in wild-type or *gim5Δ* cells exponentially growing in galactose-containing medium. The anti-Myc ChIP procedure is described in the Materials and methods section. Western blot experiments were performed in order to rule out any significant influence of the *gim5Δ* mutation on the Pfd1-Myc level. **B.** Gim5-Myc binding to *GAL1* does not depend on Pfd1. Gim5-Myc occupancy of *GAL1* was analyzed in wild-type or *pfd1Δ* cells exponentially growing in galactose-containing medium. Western blot experiments were performed in order to rule out any increase of Gim5-Myc level in the *pfd1Δ* mutant. **C.** The profile of Pfd1 binding to the transcribed region parallels Ser2-phosphorylation of the Rpb1 CTD. Occupancy of total (Rpb3), Ser5-phosphorylated (Se5-P) and Ser2-phosphorylated RNA polymerase II (Ser5-P) was analyzed across *GAL1* under activating conditions. ChIP experiments were performed using the conditions and antibodies described in the Materials and methods section. **D.** Prefoldin recruitment to the transcribed region is diminished in a *ctk1Δ* mutant. Pfd1-Myc occupancy of *GAL1* was analyzed by ChIP in wild-type or *ctk1Δ* cells exponentially growing in galactose-containing medium. In all panels, the results shown represent the mean and the standard deviation of three biological replicates.

The difference in transcription-dependent binding among the prefoldin subunits, which correlate with the GLAM phenotype of the corresponding mutants (Figure 4E), suggests the existence of a prefoldin complex variant formed by Pfd1, Gim1, Gim3 and Gim5, which would be involved specifically in transcription elongation. Among these subunits, only Gim5 was of the alpha type. We tested the binding of Pfd1 to *GAL1* in a *gim5Δ* background to find that the ChIP signal in galactose significantly reduced (Figure 5A), indicating that the binding of Pfd1 to transcribed chromatin is stabilized within the prefoldin complex. In contrast, the absence of Pfd1 did not diminish the binding of Gim5 to *GAL1* (Figure 5B), indicating that this alpha-type subunit can be recruited on its own to transcribed genes.

Pfd1 binding to *GAL1* increased with the distance to the transcription start site and reached a maximum between positions 200 and 700 (Figure 4C). Similar results were obtained with Gim5 (Figure S4C). This Pfd1 binding did not match the profile of the RNA polymerase II molecules that are phosphorylated in the Ser5 residues of the CTD domain, but closely followed the profile of Ser2-phosphorylated RNA polymerase II (Figure 5C). In contrast to Ser5 phosphorylation, equally associated with the initiation and elongation steps of transcription, Ser2 phosphorylation exclusively links to transcription elongation [18]. In order to investigate whether Ser2P is involved in the recruitment of prefoldin to transcribed genes, we repeated the experiment in a *ctk1Δ* mutant, lacking the main kinase of the CTD Ser2 residues [46]. We found that Pfd1 binding to *GAL1* was impaired by the *ctk1Δ* mutation, which occurred in parallel to the reduction in Ser2 phosphorylation exhibited by this mutant (Figures 5D and S5A). However, we were unable to detect a physical interaction between prefoldin and Ser2-phosphorylated RNA polymerase II by co-immunoprecipitation (Figures S5B,C).

Altogether, the results we present above support the existence of a specialized nuclear prefoldin capable of acting at transcription sites during elongation, whose recruitment would be favored by the Ser2 phosphorylation of the RNA polymerase II CTD.

### Prefoldin prevents RNA polymerase II arrest in long and TATA-containing genes

**Figure 6:**
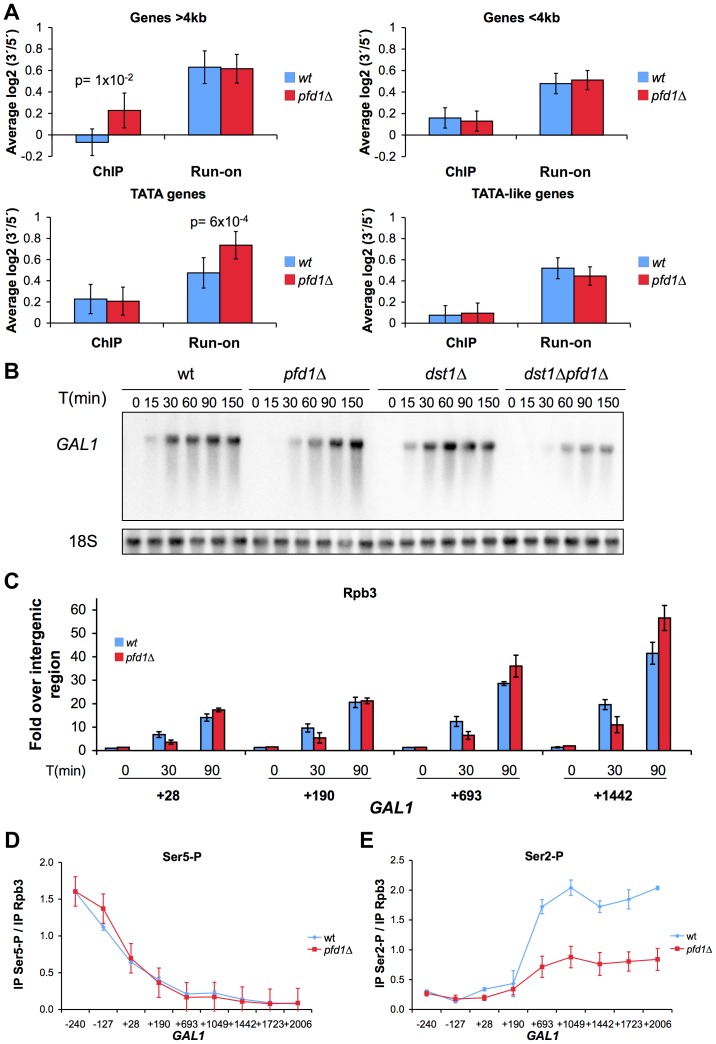
The absence of Pfd1 causes alterations in the intragenic distribution of RNA polymerase II. **A.** Comparative distribution of total RNA polymerase II (as measured by Rpb3 ChIP) and active transcription (as measured by run-on) across genes, expressed as 3′/5′ ratios. A large set of highly expressed genes were investigated in *pfd1Δ* and compared to the wild type, as described in the Materials and methods section. Average results for the specific group of genes are shown according to their length (longer or shorter than 4 kbp) or promoter type (exhibiting a canonical TATA box or a TATA-like box). Any P-values of the Student's t-test lower than 0.05 are shown. Error bars indicate standard errors. **B.** Time courses reflecting the induction of the *GAL1* gene. Cells of the indicated isogenic strains were grown in glycerol-lactate medium for two generations and then 2% galactose was added to the cultures. RNA samples were then taken at the indicated times. A significant Northern experiment is shown. The quantification of three biological replicates is shown in Figure S6A. **C.** The absence of Pfd1 alters the kinetics of RNA polymerase II occupancy during *GAL1* induction. RNA polymerase II occupancy was measured by anti-Rpb3 ChIP, after the addition of galactose to wild-type or *pfd1Δ* cells grown in glycerol-lactate medium, at the indicated times. Values correspond to the mean and the standard deviation of three biological replicates. **D.** The absence of prefoldin does not affect Ser5-phosphorylation of RNA polymerase II. The levels of Ser5-phosphorylated Rpb1 across *GAL1* were analyzed by ChIP in wild-type or *pfd1Δ* cells exponentially growing in galactose. Values were normalized to total RNA polymerase levels, as measured by anti-Rpb3 ChIP performed with the same extracts. Values correspond to the mean and the standard deviation of three biological replicates. **E.** The absence of prefoldin decreases Ser2-phosphorylation of RNA polymerase II. The levels of Ser2-phosphorylated Rpb1 across *GAL1* were analyzed and normalized as in D. Values correspond to the mean and the standard deviation of three biological replicates.

In order to evaluate the actual importance of prefoldin in transcription elongation across the genome, we measured the effect of *PFD1* deletion on the intragenic distribution of elongating RNA polymerase II. We calculated the ratios between elongating RNA polymerase II sitting on the 5′ and the 3′ ends of 377 highly expressed genes in *pfd1Δ*. We did it by following two different methods: transcriptional run-on, detecting active elongation-competent polymerases; and anti-Rpb3 ChIP, detecting all polymerases, either active or inactive [47]. When all the analyzed genes were averaged, we observed no significant change in the 3′/5′ratios in the *pfd1Δ* mutant as compared to the wild type by both ChIP and run-on (not shown). In light of the GLAM phenotype of the prefoldin mutants, we compared long genes (>4 kbp) with shorter genes. We found that long genes exhibited significantly higher 3′/5′ ratios of total RNA polymerase II (Rpb3 ChIP) in *pfd1Δ*, but this relative enrichment in 3′ did not involve any global variation in their average 3′/5′ run-on ratio (Figure 6A).

We also discovered that those genes whose promoter regions contained a canonical TATA box exhibited significantly higher 3′/5′ run-on ratios in *pfd1Δ*, with no parallel change noted in their 3′/5′ Rpb3 ChIP ratios (Figure 6A). In contrast, TATA-like genes, containing a non-canonical TATA box [48], did not undergo any significant change in *pfd1Δ* (Figure 6A). We conclude that prefoldin is required especially for transcription elongation through long and TATA genes, and that the absence of Pfd1 causes alterations in both the intragenic distribution of RNA polymerase II and its tendency to become arrested (run-on incompetent molecules).

Since *GAL1* is a canonical TATA gene, and as we detected a significant transcription-dependent binding of prefoldin to it, we investigated its transcriptional induction in *pfd1Δ*. We found a serious delay in *GAL1* mRNA accumulation in the absence of Pfd1. While the wild type accomplished 75% of induction in 30 min, *pfd1Δ* barely reached 20% (Figures 6B and S6A). This delay in mRNA accumulation was due to a transcriptional defect as it correlated with the slower occupancy of the *GAL1* coding region by RNA polymerase II, as measured by ChIP (Figure 6C). We also measured *GAL1* induction in *pfd1Δ dst1Δ* and found an even longer delay in this double mutant, which was unable to reach the wild-type level of maximum mRNA accumulation, even after 150 min (Figures 6B and S6A). In contrast, the single *dst1Δ* mutant exhibited a wild-type kinetics of induction (Figures 6B and S6A). The fact that the double mutant showed a more serious defect than any of the single mutants confirms the functional cooperation between prefoldin and TFIIS that we detected with the GLAM assays.

As mentioned above, CTD phosphorylation is a good marker of active transcription. We analyzed the levels of Ser5 and Ser2 phosphorylation of the RNA polymerase II molecules when transcribing *GAL1* in the absence of Pfd1. We detected no effect of *pfd1Δ* on the levels of Ser5 phosphorylation (Figure 6D), but we observed that the phosphorylation levels of Ser2 clearly lowered (Figure 6E). This reduction in Ser2 phosphorylation is similar to the effect caused by the deletion of the genes encoding the Paf1 complex, whose involvement in chromatin modifications during transcription elongation is well known [49].

**Figure 7:**
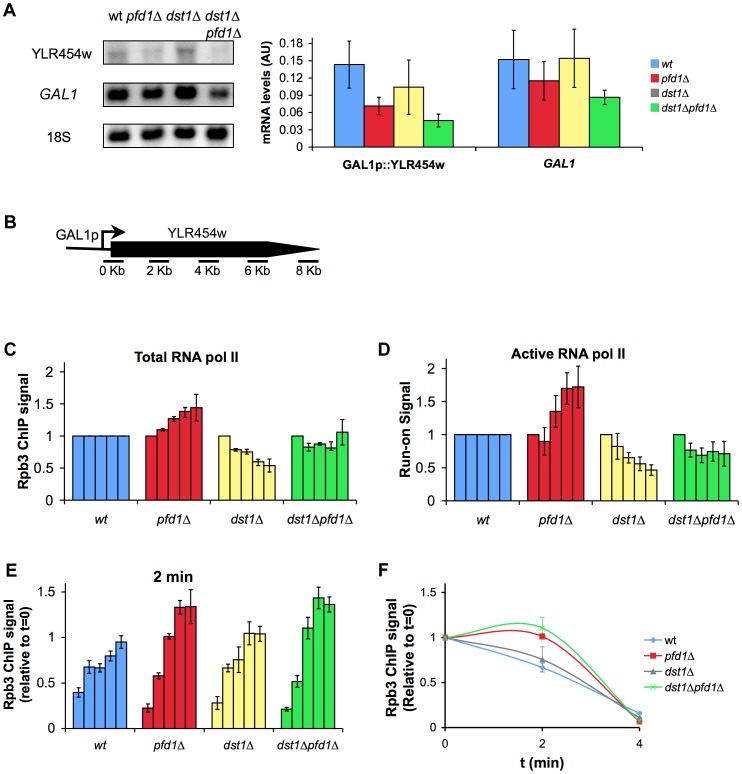
The absence of Pfd1 alters RNA polymerase II elongation across *GAL1*::YLR454w. **A.** mRNA levels of GAL1p-YLR454w are more intensively affected than those of *GAL1* in *pfd1Δ*. GAL1p-YLR454w and *GAL1* mRNA levels were measured by Northern in cells growing exponentially in galactose, as described in the Materials and methods section. A significant blot and the quantification of three biological replicas are shown. **B.** Distribution of the amplicons and probes utilized to measure RNA polymerase II occupancy across *GAL1p*-YLR454w. **C.** The profile of total RNA polymerase II along GAL1p-YLR454w is biased towards the 3′ end in *pfd1Δ*. Occupancy of RNA polymerase II was measured by anti-Rpb3 ChIP in the indicated isogenic strains. Each bar represents one of the amplicons, from 5′ (left) to 3′ (right), described in B. Values were normalized to the 5′ amplicon and to the wild type. The mean and the standard deviation of three biological replicates are shown. Non-normalized data are shown in Figure S6B. **D.** The profile of active RNA polymerase II along GAL1p-YLR454w is biased towards the 3′ end in *pfd1Δ*. Distribution of transcriptionally active RNA polymerase II along the GAL1p-YLR454w transcription unit was measured by transcriptional run-on. Cells of the indicated isogenic strains, exponentially growing in YPGAL, were tested for the presence of transcriptionally active RNA polymerases by transcriptional run-on, as it is described in the Materials and methods section. Each bar represents one of the amplicons, from 5′ (left) to 3′ (right), described in B. Values were normalized to the first probe and to the wild type. The mean and the standard deviation of three biological replicates are shown. Non-normalized data are shown in Figure S6C. **E.** The elongation rate of RNA polymerase II in GAL1p-YLR454w is affected by the absence of Pfd1. RNA polymerase II distribution is shown, 2 min after adding 2% glucose to cells of the indicated isogenic strains that were exponentially growing in galactose medium. Each bar represents one of the amplicons, from 5′ (left) to 3′ (right), described in B. RNA polymerase II levels were measured by anti-Rpb3 ChIP. Values were normalized to time 0 and correspond to the mean and the standard deviation of three biological replicates. **F.** Relative levels of RNA polymerase II occupancy of the 4 kb region of GAL1p-YLR454w during the last wave of transcription, in the indicated isogenic strains. The plot represents the time course of the normalized values of RNA polymerase II occupancy, measured by anti-Rpb3 ChIP, upon the addition of glucose to cells growing in galactose medium.

According to the data represented in Figure 6A, the maximal contribution of prefoldin to transcription elongation should take place in long, canonical TATA genes. In fact, the only three TATA genes that are longer than 4 kbp and present in the 5′/3′ array (YPL082c, YCR089w and YLR342w) exhibited increased average 3′/5′ ratios at both the total (Rpb3 ChIP) and active (run-on) RNA polymerase II levels (not shown). We analyzed in detail the effect of *pfd1Δ* on the transcription of a long transcription unit driven by a TATA promoter. We chose *GAL1p*-YLR454w, an engineered version of this 8 kbp-long gene fused to the promoter region of *GAL1* [50]. The mRNA levels expressed in galactose-containing medium confirmed that GAL1p-YLR454w exhibited greater dependency on prefoldin than *GAL1*. The accumulation of YLR454w mRNA was significantly impaired in *pfd1Δ* and in the double *dst1Δpfd1Δ* mutant (Figure 7A).

We also found a shift in the distribution of total RNA polymerase II towards the 3′ end of the gene in *pfd1Δ* in relation to the wild type (Figures 7B,C). We detected an even stronger shift of the run-on signal to the 3′ end in the absence of Pfd1 (Figure 7D). These results may well reflect a combination of the transcriptional defects caused by *pfd1Δ* on long genes and on TATA-containing genes. When we analyzed the effect of *dst1Δ* on the RNA polymerase II distribution along *GAL1p*-YLR454w, we found the opposite to be true if compared to *pfd1Δ*: reduced occupancy of Rpb3-ChIP and run-on signals towards the 3′ end of the gene (Figures 7C,D). The double *pfd1Δ dst1Δ* mutant showed flat patterns, which is in agreement with an additive and non-epistatic interaction between the two genes. All the mutants tested displayed reduction in the absolute RNA polymerase II levels (Figures S6B,C). This reduction was especially severe in the density of active RNA polymerase II molecules of the double mutant (Figure S6C) and was consistent with a more marked defect in transcription elongation when both prefoldin and TFIIS were absent.

Could TFIIS help solve those RNA polymerase arrest events caused by the absence of Pfd1? To test this hypothesis, we measured TFIIS recruitment to GAL1p-YLR454w in a *pfd1Δ* background. We observed no enrichment of TFIIS in relation to Rpb3 in *pfd1Δ* (Figure S6D). So the absence of Pfd1 interferes with elongation but it does not seem to enhance the recruitment of TFIIS. These data are consistent with a role of prefoldin in transcription elongation that is independent of TFIIS action.

Increased cryptic transcription, as described for a large set of transcription-related mutants [51], might explain the accumulation of RNA polymerase II at the 3′end of GAL1p-YLR454w (Figures 7C,D). To test this possibility, we ran a Northern blot analysis of *GAL1p*-YLR454w. We did not detect different YLR454w mRNA patterns when hybridizing with 5′ and 3′ probes (Figure S6E). Moreover, we did not detect the characteristic 5.3 kb cryptic mRNA that is transcribed from YLR454w in other mutant backgrounds [52](Figure S6E).

Finally, we investigated the elongation rate of RNA polymerase II along this transcription unit in *pfd1Δ* by shifting cells to glucose conditions. By doing so, the *GAL1* promoter switched off, so we could follow the last wave of RNA polymerase II across the gene [50]. Two minutes after glucose addition, the pattern of RNA polymerase II occupancy in *pfd1Δ* and in the double mutant reflected a slower speed of RNA polymerase II as compared to the wild type and to the single *dst1Δ* mutant. This reduction in speed was particularly visible in the 3′ half of the gene, occupied at this time by the front of the last wave of transcription (Figures 7E,F). This result is consistent with a positive effect of prefoldin in the elongation rate of RNA polymerase II. Curiously, when we measured the distribution of RNA polymerase II 4 min after switching the promoter off, we found a pattern that did not indicate a slower speed, but rather a slightly faster movement of the last subpopulation of RNA polymerase II molecules; that is, the tail of the last wave of transcription (Figures 7F, S6F) (see Discussion).

### Prefoldin enhances histone dynamics during transcription elongation

Several previous works have reported genetic interactions between the prefoldin subunits and the factors involved in the transcriptional dynamics of chromatin that have never been specifically addressed (Table 1). For example, negative genetic interactions, involving poorer growth of double mutants than what is expected from the growth of the corresponding single mutants, have been detected between prefoldin subunits and the following chromatin factors: Set2 [30]; H2AZ and the SWR complex involved in the H2AZ/H2A exchange [53]–[55]; the histone deacetylase-containing Set3 and Rpd3 complexes (Collins et al, 2007); the histone acetylase-containing NuA4 complex [54], [55]; the Mediator complex [54]; and the ATP-dependent nucleosome remodeling complexes SWI-SNF [55], RSC [54] and ISW [54].

**Table 1:** Genetic interactions between prefoldin and chromatin factors.

Factor	Type of interaction	Reference
Set2	Negative	[30]
H2AZ, SWRC	Negative	[30], [54], [55]
NuA4	Negative	[54], [55]
Set3C	Negative	[54]
Rpd3C	Negative	[54]
Mediator	Negative	[54]
SWI/SNF	Negative	[55]
RSC	Negative	[54]
ISW	Negative	[54]
PAF1C	Positive	[54], [55]
Bre1-Lge1	Positive	[54]
Set1C	Positive	[54]
Chd1	Positive	[54], [55]
SAGA	Positive	[54]

Positive genetic interactions, involving stronger growth of double mutants than what is expected based on the growth of the single mutants, were detected between the prefoldin subunits and Chd1 [54], [55], the SAGA complex [54], the PAF1 complex [54], [55], the Bre1-Lge1 complex [54] and the Set1 complex [54]. Three of the complexes exhibiting positive interactions with prefoldin cooperate in a sequence of histone modifications events that involves H2B ubiquitination (PAF1C, Bre1-Lge1) and H3-K4 trimethylation (Set1C) [56], [57]. The other two (SAGA and Chd1) copurify and link H3-K4 methylation to histone tails acetylation [58].

**Figure 8:**
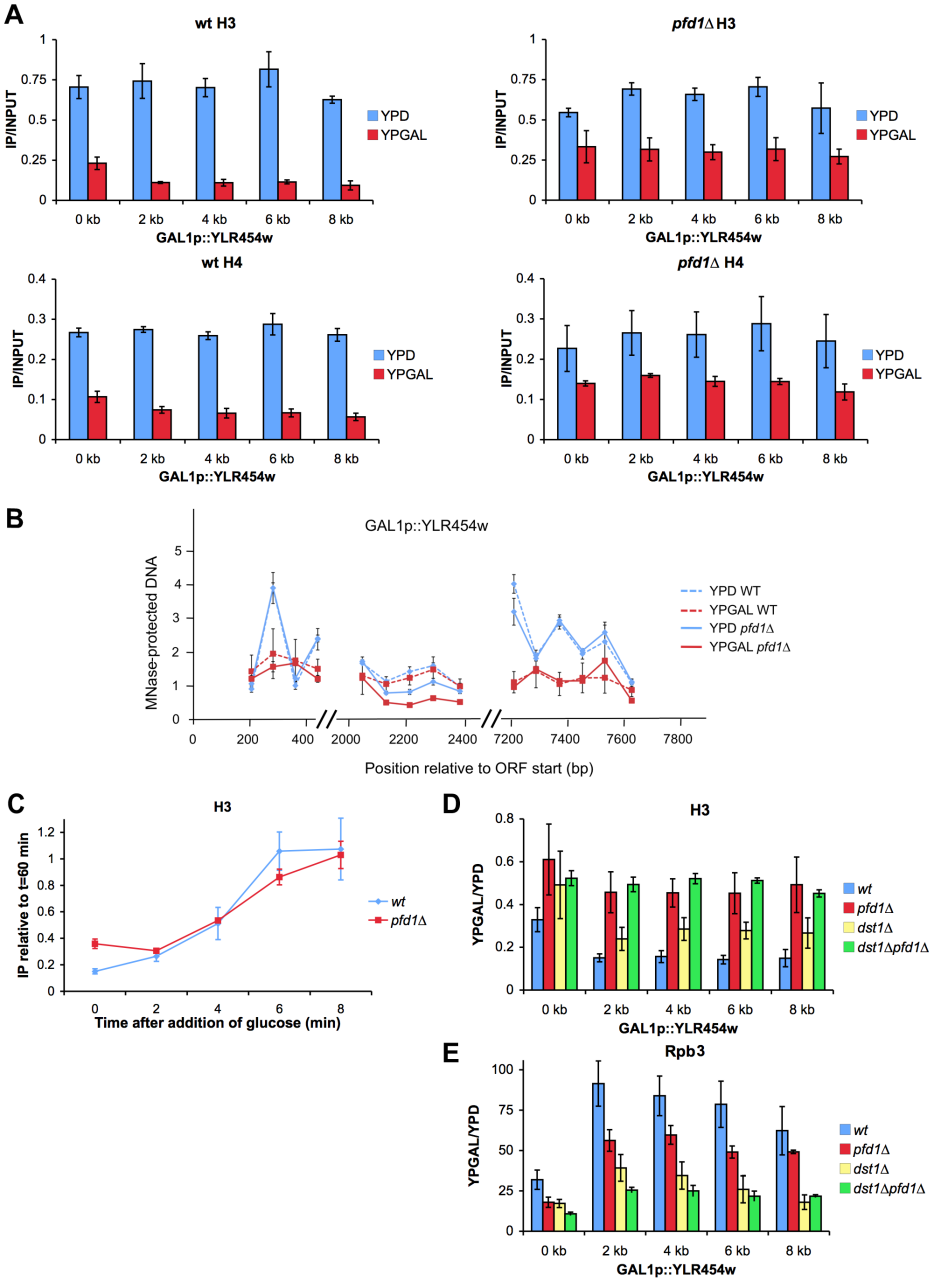
Histone dynamics during transcription elongation is impaired in *pfd1Δ*. **A.** The absence of Pfd1 impairs the characteristic difference in histone occupancy along the transcribed region that occurs between active and inactive genes. Binding of H3 and H4 to transcribed (YPGAL) and untranscribed (YPD) GAL1p-YLR454w was measured by ChIP in wild-type (left) and *pfd1Δ* (right) exponentially growing cells. Values correspond to the mean and the standard deviation of three biological replicates. **B.** Sensitivity of GAL1p-YLR454w chromatin to micrococcal nuclease, under activating (YPGAL) and non activating (YPD) conditions is not affected by the absence of Pfd1. Note that the characteristic up and down pattern caused by the positioned nucleosomes present in the 5′ and 3′ ends of the gene under repressed conditions is equally absent in the wild type and in *pfd1Δ*. Cells exponentially growing in the indicated media were permeabilized and treated with MNase, as described in the Materials and methods section. Mononucleosomal DNA was then extracted and analyzed by quantitative PCR. Values were normalized to naked DNA digested with MNase and processed in parallel to the chromatin samples. Values correspond to the mean and the standard deviation of two biological replicates. **C.** The kinetics of the increase in histone occupancy that occurs after transcriptional repression is not influence by the absence of Pfd1. Histone occupancy in the 4 kb region of GAL1p-YLR454w was analyzed during the last wave of transcription after switching the *GAL1* promoter off. Glucose was added at time 0 to wild-type and *pfd1Δ* cells growing exponentially in galactose. At the indicated times samples were taken to measure H3 binding to the 4 kb region by ChIP. Values were normalized to the levels of H3 occupancy measured 60 min after the addition of glucose to the cultures. The mean and the standard deviation of three biological replicates are shown. **D and E.** The difference in H3 occupancy produced by *pfd1Δ* and *dst1Δ* across GAL1p-YLR454w does not correlate with their effect on RNA polymerase II occupancy. Ratios of H3 occupancy between YPGAL and YPD in the indicated mutants across GAL1p-YLR454w (D), and levels of RNA polymerase II bound to GAL1p-YLR454w (E). H3 and Rpb3 occupancy was measured by ChIP, as described in the Materials and methods section. Values correspond to the mean and the standard deviation of three biological replicates.

The genetic interactions described above suggest the functional relevance of prefoldin in histone dynamics during transcription. We evaluated this hypothesis by measuring histone occupancy on *GAL1p*-YLR454w. In wild-type cells, occupancy of the whole transcribed region by histones H3 and H4 diminished drastically under activating conditions (Figure 8A), as previously reported [44]. *pfd1Δ* exhibited only a minor decrease in histone occupancy, when cells were grown in glucose-containing medium, if compared to the wild type (Figure 8A). However, in galactose medium, we observed significantly higher levels of bound H3 and H4 in *pfd1Δ* than in the wild type (Figure 8A). In the latter, histone occupancy was 3-fold (5′ end) or 5-fold (the remaining amplicons) lower in galactose than in glucose. Unlike the wild type, these ratios in *pfd1Δ* ranged between 1.5 and 2 (Figure 8A). Similar results were obtained with histone H2B (Figure S7A). Milder, yet still significant effects of *pfd1Δ* on histone occupancy in galactose were detected in *GAL1* (Figure S7B).

We investigated whether the excess H3 detected across GAL1p-YLR454w in *pfd1Δ* was trimethylated in the K36 residue (H3K36Me3) because this modification is closely associated with transcription elongation [30]. We found that the absolute levels of H3K36Me3 across GAL1p-YLR454w were higher in *pfd1Δ* than in the wild type (Figure S7C), indicating that at least a fraction of excess H3 accumulated in *pfd1Δ* had undergone methylation. This fact supports the notion that the higher H3 occupancy detected in *pfd1Δ* is not just due to lower transcriptional activity. When we normalized H3K36Me3 levels to total H3, we found it lower in *pfd1Δ* than in the wild type (Figure S7D), indicating that another fraction of excess H3 is not methylated in K36. This transcribed unmethylated H3 is likely the consequence of the decreased level of CTD-Ser2 phosphorylation caused by *pfd1Δ* (Figure 6E), which should affect the recruitment of the methylase Set2 [16]. However, the overall effect of *pfd1Δ* on H3K36 methylation is quantitatively limited since the global cellular levels of H3K36Me3 did not change significantly in the mutant (Figure S7E).

The accumulation of histones that we found across GAL1p-YLR454w in *pfd1Δ* under activating conditions can be explained by a defect in nucleosome remodeling. To test this hypothesis, we analyzed nucleosome remodeling by measuring the sensitivity of three different regions (5′, middle and 3′) of GAL1p-YLR454w to micrococcal nuclease (MNase). We found typical nucleosomal peaks in 5′ and 3′ under repressive conditions (Figure 7B). In these two regions, DNA sensitivity to MNase was significantly greater under activating conditions (Figure 7B). No peaks were detected in either glucose or galactose, in a central region of the gene that does not exhibit positioned nucleosomes (Figure 7B). No significant difference was found between *pfd1Δ* and the wild type in any of the three regions of the gene. Similar results were obtained for the 3′ region of *GAL1* (Figure S7F). We conclude that prefoldin is not required for the initial nucleosome-remodeling step, which destabilize DNA-octamer interactions and facilitate nucleosome mobility upon transcription activation.

The higher levels of histone occupancy across the transcribed GAL1p-YLR454w gene in *pfd1Δ* could be due to increased efficiency in chromatin reassembly or to impaired histone eviction. To test the first possibility, we analyzed the kinetic of histone reassembly after switching the promoter off. H3 ChIP signals were quantified in the central region (4 kb amplicon) of GAL1p-YLR454w at different times after adding glucose to cells exponentially growing in galactose medium. We found no significant difference in the kinetics of H3 occupancy when comparing *pfd1Δ* and the wild type (Figure 8C). If any, a slightly slower rate was detected in the prefoldin mutant. Since neither nucleosome remodeling nor chromatin reassembly seems to be affected by *pfd1Δ*, we propose a specific role for prefoldin in histone eviction during transcription elongation.

Inverse correlation between histone occupancy and RNA polymerase II density has been reported [59], [60]. Therefore, the defect in histone eviction that we observed in *pfd1Δ* might merely be an indirect consequence of suboptimal transcription caused by a different molecular defect. The higher absolute levels of H3K36Me3 (Figure S7C) and the normal nucleosome-remodeling pattern exhibited by GAL1p-YLR454w in *pfd1Δ* (Figures 8B and S7F) contradict this hypothesis. Nevertheless, we tested it by measuring H3 and H2B occupancy in *dst1Δ* and in the double mutant *pfd1Δ dst1Δ* as both showed significantly lower levels of RNA polymerase II across GAL1p-YLR454w than *pfd1Δ* (Figure S6B). In *dst1Δ*, we found much less excess in histone occupancy than in *pfd1Δ* (Figures 8D and S7G). In the double mutant, which exhibited stronger transcriptional deficiency than *pfd1Δ*, we observed the same excess in histone occupancy as in *pfd1Δ* (Figures 8D and S7G). Hence, we found no kind of correlation between the effect of a mutation on the occupancy of the transcribed gene by histones and by RNA polymerase II. What we did observe was a clear correspondence between the *pfd1Δ* genotype and the retention of histones under activating conditions (Figures 8D,E and S7G). Taken altogether, the above data are compatible with a functional contribution of prefoldin to histone eviction during transcription elongation.

## Discussion

We applied the systematic genetic analysis of yeast deletions to the study of transcription elongation by making full use of the GLAM assay. Genetic analysis is a powerful tool to detect the involvement of specific genes in a given biological function. It proves especially useful to identify unexpected functional links between already known cellular elements and biological processes not related to date. This is the case of the novel role of prefoldin in transcription elongation that we have uncovered.

The involvement of prefoldin in the cytoplasmic folding of cytoskeletal components is well established [25], [26]. Prefoldin-like complexes are also involved in the cytoplasmic assembly of eukaryotic RNA polymerase II [61]. Accordingly, the transcriptional defects of prefoldin mutants might result indirectly from its cytoplasmic role. This does not seem to be the case, as we detected the presence of prefoldin inside the nucleus and its binding to the coding region of expressed genes in a transcription-dependent manner. Moreover, the chromatin binding and transcriptional contribution among prefoldin subunits closely correlate, whereas at least one prefoldin mutant (*pfd1Δ*) displays clear transcriptional defects without exhibiting any significant cytoskeletal dysfunction. Likewise, we detected no impairment in transcription elongation upon treatment with the microtubule-destabilizing drug benomyl. We, therefore, consider that the transcriptional defects exhibited by prefoldin mutants reflect a relevant nuclear role of prefoldin in transcription elongation.

Nuclear prefoldin might work together with nuclear actin, which contributes to transcription elongation in metazoa [62] [63]. However, recruitment of actin to transcribed loci is maximal in the promoter region and its binding decreases progressively along genes [64], whereas the prefoldin profile peaks in the 3′ end of genes in parallel to the Ser2-phosphorylation of RNA polymerase II-CTD. Moreover, *pfd1Δ* is not hypersensitive to latrunculin A, which is able to disrupt actin filaments. This suggests that, even if prefoldin acts on nuclear actin, there is another nuclear role of prefoldin in transcription elongation.

Prefoldin subunits have been connected to other nuclear events where actin does not seem to be involved, like the inhibition of the c-Myc transactivation domain [65] and the removal of HIV integrase before viral transcription [66]. The regulated migration of prefoldin from the cytoplasm to the nucleus, where it interacts with DELLA transcription factors, has been recently described in plants [67]. Our experimental results complete this nuclear perspective of prefoldin and establish a functional connection between prefoldin and transcription elongation.

A direct action of prefoldin on the elongating form of RNA polymerase II or in RNA polymerase II clearance after termination might explain the recruitment of prefoldin to gene bodies and the accumulation of hypophosphorylated RNA polymerase II at 3′ in its absence. According to this hypothesis, the effect of prefoldin on histone eviction would be indirectly mediated by RNA polymerase II. This would be supported by the described interactions of prefoldin-like complexes with human RNA polymerase II during their cytoplasmic assembly [61]. However, the prefoldin subunits involved in human RNA polymerase II assembly (Gim2 and Gim4) do not exhibit transcriptional phenotypes in yeast (Figure 2B). Moreover, we failed to detect any physical interaction between prefoldin and elongating RNA polymerase II by co-immunoprecipitation (Figure S5B).

Although further work will be necessary to elucidate the direct target of prefoldin in the transcription sites, the evidence presented in this article rather indicates prefoldin to be an effector of chromatin dynamics during transcription elongation. This role explains the large set of genetic interactions linking prefoldin and chromatin factors (summarized in Table 1). We detected defects in co-transcriptional histone eviction in *pfd1Δ* and we showed that they were not merely due to suboptimal transcriptional activity. In fact, we detected optimal accessibility of micrococcal nuclease to transcribed DNA in the prefoldin mutant, suggesting that the initial steps of cotranscriptional chromatin dynamics are not impaired in the absence of prefoldin (Figure 8B, S7F). Likewise, we did not detect a positive effect of the *pfd1Δ* mutation on chromatin reassembly after the passage of RNA polymerase II (Figure 8C). Taken altogether, these results are fully compatible with a role of prefoldin in histone eviction during transcription elongation. This would imply that histone eviction is not an automatic consequence of nucleosome remodeling, but an independent step requiring specific factors. In line with this, prefoldin would not be unique. The mutation of other chromatin-related factors that stimulate transcription elongation, like the histone acetyl transferases Esa1 and Gcn5, also dampen cotranscriptional histone eviction and provoke gene length-dependent defects in transcription [44], [68]. In this scenario, the accumulation of hypophosphorylated RNA polymerase II across the transcribed region would be the consequence of impaired chromatin dynamics, rather than the primary effect of the prefoldin absence. In turn, decreased CTD-Ser2 phosphorylation would additionally impact chromatin dynamics by reducing the proportion of H3 that undergoes K36 methylation [16].

According to our results, two mutants both causing decrease in the RNA polymerase II occupancy of gene bodies (*dst1Δ* and *pfd1Δ*) exhibit clearly different levels of bound histones (Figures 8D,E and S7G). This fact is compatible with the existence of alternative modes of chromatin dynamics during transcription elongation, as previously suggested [69] [70]. After nucleosome remodeling, histones might be either evicted by specific machinery, which includes prefoldin, or rearranged within the remodeled octamer (Figure S8). Accordingly, *in vitro* experiments have shown that RNA polymerase II can transcribe a partially disassembled octamer, the hexasome, without the simultaneous eviction of all histones [71]–[73]. According to our results, these two modes of chromatin dynamics during elongation can operate in any gene but their relative preponderance would be gene-specific depending on characteristics like length and promoter type. The differential effect of *pfd1Δ* on the front and the tail of the last wave of transcription, as we detected in the GAL1p-YLR454w system (Figures 7E,F and S6F), might reflect these two alternative modes of elongation through chromatin.

The existence of alternative modes of dealing with chromatin during elongation predicts negative synthetic phenotypes for those elongation factors that play their role in different pathways. TFIIS and prefoldin indeed show very severe synthetic impairment in all the transcriptional phenotypes that we explored. The preferential participation of TFIIS in the hexasome mode of transcription, where RNA polymerase II would display a greater tendency to backtrack, would explain this negative interaction (Figure S8).

The set of genetic interactions between prefoldin and chromatin factors is similar to that described for the CCT complex; that is, the chaperonin that cooperates with prefoldin in its cytoplasmic function [74]. It is feasible, therefore, that prefoldin and CCT also cooperate in their chromatin task. Another protein chaperon, Hsp90, also plays an important role in gene transcription by stabilizing negative elongation factor NELF on paused elongating RNA polymerase II [75]. Altogether, these findings situate chaperone-related factors as important players in gene regulation.

## Materials and Methods

### Genetic screening

We introduced the plasmids of the GLAM system [28] into the library of *S. cerevisiae* viable deletion mutants using the method previously described for SGA [76]. In short, plasmids pSCh202 (pGAL1-PHO5) and pSCh212 (pGAL1-PHO5-lacZ) were transformed into carrier strain BY5563 (*MAT alpha*). The transformants obtained were then mated to the deletion library (*MAT a*) and sporulated for 7 days. The haploid spores carrying the plasmid (pSCh202 or pSCh212) and the deletion mutation were selected in the appropriate media.

The resulting strains were then inoculated in duplicates into 96-well plates containing 200 µl of SGal-Ura medium per well, and were incubated for 48 h at 30°C before assaying acid phosphatase activity as described elsewhere [28]. The GLAM ratio was calculated as the phosphatase activity in the deletion strain carrying pSCh212 divided by the activity in the corresponding strain bearing pSCh202. The experiment was performed in two independent biological replicates with two technical replicates in each.

### Strains, plasmids and growth conditions

The strains and plasmids used in this study are listed in Table S2. All the *S. cerevisiae* strains utilized were in the BY4741 background, except for the *xpo1-1 mex67-5* strain, which was in the W303 background. Strains were grown at 30°C in the indicated media, except for the *xpo1-1 mex67-5* mutant, which was shifted from 24°C to 37°C for 4 h.

To generate pGMZ2, a PCR product containing the PFD1 ORF lacking the termination codon and an additional 1 kb upstream of the ORF was obtained by PCR using the oligonucleotides listed in Table S3 and yeast genomic DNA as a template. This product was cloned in pGEM-T easy (Promega), to be then restricted and cloned into YCplac33-yGFP. pGMZ3, pGMZ4, pGMZ5, pGMZ6 and pGMZ7 were generated similarly. In all cases, GFP constructs complemented the null allele to the wild-type extent. Absence of GLAM phenotype and sensitivity to benomyl were checked.

To generate Pfd1-Myc5, the Y07202 yeast strain was transformed with a PCR product obtained using the oligonucleotides listed in Table S3 and pGA2266 plasmid DNA as a template. Transformants were selected in SC-TRP and checked by PCR and Western Blot. Gim1-Myc, Gim2-Myc, Gim3-Myc, Gim4-Myc and Gim5-Myc were generated similarly. Absence of the GLAM phenotype and sensitivity to benomyl were verified.

### Growth assays

For the growth assays, yeast cultures were diluted to OD_600_ 0.5 and serial dilutions (1∶10) were spotted onto plates. The Latrunculin A-sensitivity assay was performed as previously described [77]. Mycophenolyc acid, benomyl and latrunculin A were purchased from SIGMA.

### Fluorescence microscopy

Cells were grown to the mid-log phase in selective SD liquid medium, and were washed and resuspended in TBS buffer. Image acquisition was done either with a Leica DMR microscope equipped with a differential contrast (DC) camera, or with a Zeiss LSM 710 laser scanning confocal microscope at 2 µm pinhole aperture. Digital images were processed with Adobe Photoshop CS.

### RNA analysis

Six micrograms of total RNA prepared from yeast cells were subjected to electrophoresis on formaldehyde agarose gels, transferred to Hybond-N filters (Amersham Biosciences, UK) and UV cross-linked prior to hybridization at 65°C with a [^32^P]dCTP-labeled DNA probe. Membranes were exposed in Fuji BAS screens and developed with a FUJIX FLA5100 device. All the values were normalized in relation to the present amount of 18S rRNA.

### Protein analysis

Laemmli-boiled crude extracts were run on a 12% SDS-polyacrylamide gel and transferred to nylon membranes (Hybond-ECL). After blocking with Tris-buffered saline containing 0.1% Tween 20 and 5% milk, the following primary antibodies were used: mouse monoclonal anti-Myc (Santa Cruz Biotechnology, Inc.), rabbit polyclonal anti-L1 [54], rabbit polyclonal anti H3 (Abcam), or rabbit polyclonal anti-H3K36Me3 (Abcam). Finally, peroxidase-conjugated goat anti-mouse or goat anti-rabbit IgG (both from Bio-Rad) were used to detect the specific proteins.

### Co-immunoprecipitation experiments

Yeast nuclear extracts were obtained as previously described in [78]. Immunoprecipitation was performed using IgG Sepharose (Healthcare) or protein A Sepharose (Healthcare) following the procedure described in [79].

### Chromatin immunoprecipitation

ChIP experiments were performed as previously described [27]. Immunoprecipitations were performed with magnetic beads (Dynal) using the following antibodies: Myc (Santa Cruz Biotechnology), HA (clone 3F10, Roche) Rpb3 (ab81859; Abcam), Ser2-P (ab5095, Abcam), Ser5-P (ab5131, Abcam), H3 (ab1791, Abcam), H2B (ab1790, Abcam), H3 tri methyl K36 (ab9050, Abcam), H4 (ab10158, Abcam). DNAs were analyzed by real-time quantitative PCR using SYBR Green Premix Ex Taq (Takara) and Light Cycler 480 II (Roche) with the primers listed in Table S3.

### Pfd1 ChIP on chip

For Pfd1-Myc ChIP on chip experiments, chromatin immunoprecipitation was performed as above and, after crosslinking reversal, the obtained fragments (300 bp approximately) of enriched DNA were amplified unspecificly and labeled following Affymetrix Chromatin Immunoprecipitation Assay Protocol P/N 702238. Genomic DNA controls were processed in parallel. After PCR amplification with dUTP, the samples were purified using Qiagen QIAquick PCR Purification Kit (50) (Cat.No. 28104). DNA quality and quantity were checked using a NanoDrop ND-1000 Spectrophotometer. 0.5 µg of each were used to hybridize GeneChip S.Cerevisiae Tiling 1.0R custom arrays. This step was carried out in the Multigenic Analysis Service of the University of Valencia. The obtained CEL archives were normalized and the intensities of the signal were extracted using the TAS (Tiling Analysis Software) developed by Affymetrix. The resulting text files were read using R scripts to adjudicate probe intensities to genes. The log_2_ values of the median intensities of the chosen different group of genes were represented.

### Transcriptional run-on

Run-on assays were performed as previously described [47] with minor modifications. Basically, 50 ml of yeast culture were collected at OD_600_ 0.5 by centrifugation. Cells were washed in 5 ml of 0.5% sarkosyl solution. Cells were centrifuged and the supernatant was completely removed. The transcription reaction was incubated for 5 min at 30°C. RNA was immediately extracted following the acid-phenol protocol.

Slot-blotted membranes were performed as formerly described [47]. Double-strand immobilized probes were obtained by PCR using the primers listed in Table S3.

### Analysis of transcriptional 3′/5′ratios

DNA arrays were produced in the Sección de Chips de DNA-S.C.S.I.E of the University of Valencia as previously described [47]. Briefly, 300-bp-length double strand DNA probes of 377 ORFs were obtained by PCR and printed onto positively charged nylon membranes using a BioGrid robot (BioRobotics).

Hybridization with labeled RNA (Run-on) or DNA (Rpb3 ChIP) samples was performed as described [47]. Shortly, radiolabeled RNA from the run-on was fragmented and denatured prior to hybridization by adding NaOH to the sample. Membranes were exposed in Fuji BAS screens for 5–7 days and were developed with a FUJIX FLA30000 device. Signals were quantified using the Array Vision software, version 8.0 (Imaging Research Inc.).

Data analysis and normalizations were performed according to [47]. Only those spots with signals 1.3 times over the background were considered. In all, 270 genes were successfully analyzed, of which 44 were longer than 4 kbp and 70 contained a canonical TATA box [80]. After quality filters, the log_2_ of the 3′/5′ratio was obtained. At least three replicas of each experiment were performed.

For Rpb3 ChIP on chip purposes, the immunoprecipitated DNA was amplified and radiolabeled following the procedure described in [81].

### Chromatin remodeling analysis

Yeast spheroplasts and micrococcal nuclease digestions were performed as previously described [82]. DNAs were analyzed by real-time quantitative PCR using SYBR Green Premix Ex Taq (Takara) and Light Cycler 480 II (Roche). The chromatin/naked-DNA ratio was normalized to the *CDC10* probe described in Table S3.

## Supporting Information

Figure S1 SGA screening based on the GLAM assay. **A.** Relevant information of the two transcription units utilized to obtain the GLAM ratios. **B.** Schematic description of the process followed during screening.
(TIF)

Figure S2 Transcriptional phenotypes of prefoldin do not correlate with sensitivity to benomyl or latrunculin A. **A.** GLAM ratios obtained after incubating wild-type cells in the presence of benomyl for the indicated times. 15 µg/ml benomyl was added to wild-type cells growing exponentially in selective medium. GLAM assay was performed at the indicated times. Mean and standard deviation of 3 biological replicates are shown. **B.** Drop assay showing that *pfd1Δ* exhibits much weaker sensitivity to benomyl than the other prefoldin mutants. Serial dilutions of exponentially growing cultures of the indicated strains were plated on YPD and YPD plus 15 µg/ml benomyl and incubated during 3 days at 30°C. **C.** Plate assay showing that *gim2Δ* is hypersensitive to latrunculin A, whereas *pfd1Δ* is not. The concentration of latrunculin A soaking each position is shown. Histograms represent the diameter of the inhibition halo of the 0.5 mM position for each strain after 2 days of incubation.
(TIF)

Figure S3 Nucleo-cytoplasmic localization of prefoldin. **A.** The Pfd1-GFP fusion protein is functional. GLAM assays were performed with cells from a *pfd1Δ* transformant expressing Pfd1-GFP, from the non-transformed mutant and from an isogenic wild-type strain. The results shown represent the mean GLAM ratios and the standard deviations of three biological replicates. **B.** Analysis of cells expressing the indicated GFP fusions by confocal microscopy. The results reveal nucleo-cytoplasmic distribution for these proteins. Confocal microscopy was performed as described in the Materials and methods section. Nuclei were stained with Hoechst.
(TIF)

Figure S4 Prefoldin binds chromatin. **A.** The Pfd1-Myc fusion protein is functional. GLAM assays were performed with cells from a *pfd1Δ* transformant expressing Pfd1-Myc, from the non-transformed mutant and from an isogenic wild-type strain. The results shown represent the mean GLAM ratios and the standard deviations of three biological replicates. **B.** and **C.** Binding of Myc fusions of the indicated prefoldin subunits along *GAL1* under repressed (YPD) and activating (YPGAL) conditions. Cells exponentially growing in glucose- (YPD) or in galactose-containing medium (YPGAL) were analyzed by anti-Myc ChIP, as described in the Materials and methods section, using amplicons centered in the indicated *GAL1* positions. The results shown represent the means and the standard deviations of three biological replicates.
(TIF)

Figure S5 Recruitment of prefoldin to transcribed genes. **A.** *ctk1Δ* diminishes Ser2 phosphorylation of Rpb1 CTD. Occupancy of Ser2-phosphorylated RNA polymerase II was analyzed by ChIP using the same wild-type and *ctk1Δ* extracts of the Pfd1-Myc experiments shown in Figure 5D. The results shown represent the means and the standard deviations of three biological replicates. **B.** Co-immunoprecipitation experiments do not support a direct interaction of Pfd1 to Ser2-phosphorylated RNA polymerase II. 6 mg of concentrated yeast nuclear extract from Pfd1-TAP cells were incubated either with IgG-Sepharose or with Protein-A sepharose (negative control) during 4 h at 4°C on rotation. After washing extensively, half of the IP material was loaded in a 12% polyacrylamide SDS gel and analyzed for the presence of Pfd1-TAP by Western blot, and the other half was loaded in a 7% polyacrylamide SDS gel and analyzed for the presence of Ser2-phosphorylated Rpb1. In both cases 1% of the input material and 1% of the flow-through material were also analyzed. **C.** The Pfd1-TAP fusion protein used in the co-immunoprecipitation experiments shown above is functional. GLAM assays were performed with cells from a *pfd1Δ* transformant expressing Pfd1-TAP from the non-transformed mutant and from an isogenic wild-type strain. The results shown represent the mean GLAM ratios and the standard deviations of three biological replicates.
(TIF)

Figure S6 Transcriptional function of prefoldin. **A.** Time courses reflecting the induction of the *GAL1* gene by the addition of galactose to cells grown in glycerol-lactate medium. Cells of the indicated isogenic strains were grown in glycerol-lactate medium for two generations and then 2% galactose was added to the cultures. RNA samples were then taken at the indicated times and used for Northern experiments. Mean and standard deviation of the quantitated Northern signal of three biological replicates is shown. **B.** RNA polymerase II distribution across *GAL1p*-YLR454w. Occupancy of RNA polymerase II was measured by anti-Rpb3 ChIP in the indicated isogenic strains. Points represents the amplicons described in Figure 7B. The same data as in Figure 7C are shown, except the values here are not normalized. **C.** Distribution of transcriptionally active RNA polymerase II across *GAL1p*-YLR454w, as measured by run-on. The same data of Figure 7D are shown, except values here are not normalized. **D.** TFIIS occupancy of GAL1p-YLR454w does not increase in the absence of prefoldin, as shown by Rpb3 and TFIIS-HA ChIP experiments. Wild-type and *pfd1Δ* cells expressing the HA-tagged version of TFIIS in its N-terminus and growing exponentially in YPGAL were processed for ChIP. The same extract was used to perform Anti-Rpb3 and anti-HA ChIP, in order to calculate TFIIS/Rpb3 ratios for each amplicon. Means and standard deviations of three biological replicates are shown. **E.** Northern blots of GAL1p-YLR454w in the indicated isogenic strains, showing the absence of the 5.3 kb cryptic transcript that these genes expressed in other genetic backgrounds [52]. The blot was hybridized with 5′ and 3′ probes in order to also explore antisense cryptic transcription. 1-day (left) and 3-days (right) exposures are shown. **F.** The elongation rate of RNA polymerase II in GAL1p-YLR454w is affected by the absence of Pfd1. RNA polymerase II distribution is shown, 4 min after adding 2% glucose to cells of the indicated isogenic strains that were exponentially growing in galactose medium. RNA polymerase II levels were measured by anti-Rpb3 ChIP. Values were normalized to time 0 and correspond to the mean and the standard deviation of three biological replicates.
(TIF)

Figure S7 Prefoldin contribution to chromatin dynamics. **A.** The absence of Pfd1 impairs the characteristic difference in histone occupancy along the transcribed region that occurs between active and inactive genes. The levels of H2B bound to transcribed (YPGAL) and untranscribed (YPD) GAL1p-YLR454w were measured by ChIP in wild-type (left) and *pfd1Δ* (right) exponentially growing cells. **B.** *pfd1Δ* also impairs transcriptional histone dynamics in *GAL1*. H3 and H2B binding to *GAL1* were measured in the same experiment described in **A.** **C.** and **D.** The chromatin of the GAL1p-YLR454w transcribed region is enriched in trimethylated H3K36, although most H3 histones accumulated in transcribed GAL1p-YLR454w in *pfd1Δ* are not methylated in H3K36. The levels of H3K36Me3 bound to transcribed GAL1p-YLR454w was measured by ChIP in wild-type and *pfd1Δ* cells exponentially growing in YPGAL (C). Levels of total H3 bound to the same gene were also measured in the same extracts and used to calculate H3K36Me3/H3 ratios. Means and standard deviation of three biological replicates are shown. **E.** Overall H3K36 trimethylation does not change in the absence of Pfd1. Whole cell extracts of wild-type and *pfd1Δ* were used to analyze the cellular levels H3K36Me3 and total H3, as described in the Materials and methods section. **F.** Sensitivity of *GAL1* chromatin to micrococcal nuclease, under activating (YPGAL) and non-activating (YPD) conditions is not affected by the absence of Pfd1. The experiments were performed as described in Figure 8. **G.** Transcriptional eviction of H2B in *GALp-YLR454w*, expressed by the ratio between the ChIP signals of the cells grown in YPGAL and YPD, in the wild type and in the indicated isogenic mutants.
(TIF)

Figure S8 A model describing the potential role of prefoldin in histone dynamics during transcription elongation. Green ovals depict active RNA polymerase II; the pink one indicates backtracked RNA polymerase II. The other figures represent histones.
(TIF)

Table S1 GLAM ratios of the *Saccharomyces cerevisiae* deletion mutants. calculated as described in Materials and methods. Only consistent values, reproduced in the two biological replicates of the screening, are shown.
(XLS)

Table S2 Strains and plasmids utilized in this work.
(DOC)

Table S3 DNA oligonucleotides utilized in this work.
(DOC)
