# Supplementary figures and images for: The Prefoldin Complex Regulates Chromatin Dynamics during Transcription Elongation
Source: PLoS Genet. 2013 Sep 19;9(9):e1003776. doi: 10.1371/journal.pgen.1003776 (PMC3777993; doi:10.1371/journal.pgen.1003776)

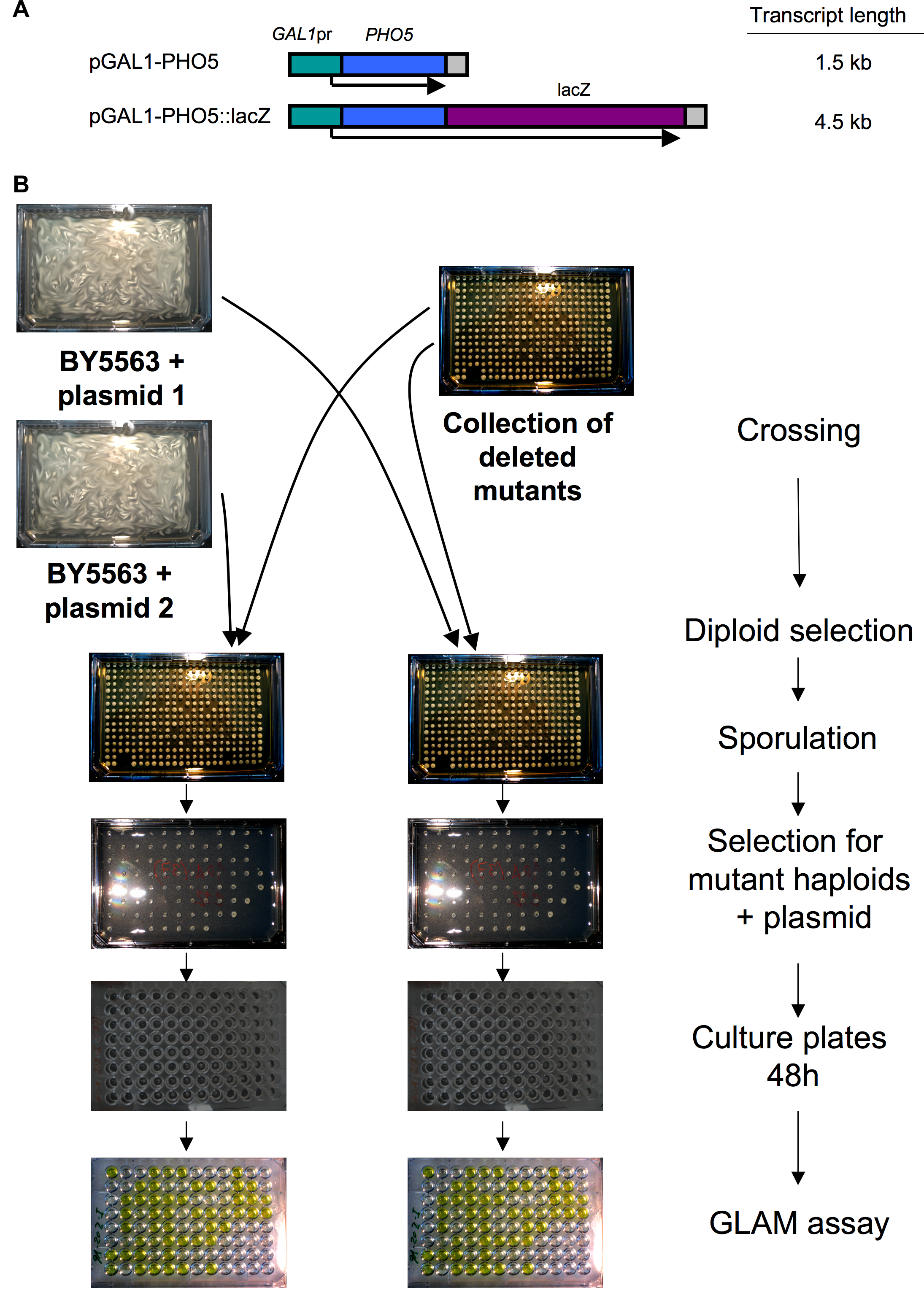

Supplement: Figure S1 — SGA screening based on the GLAM assay. A. Relevant information of the two transcription units utilized to obtain the GLAM ratios. B. Schematic description of the process followed during screening. (TIF) [file pgen.1003776.s001.tif]

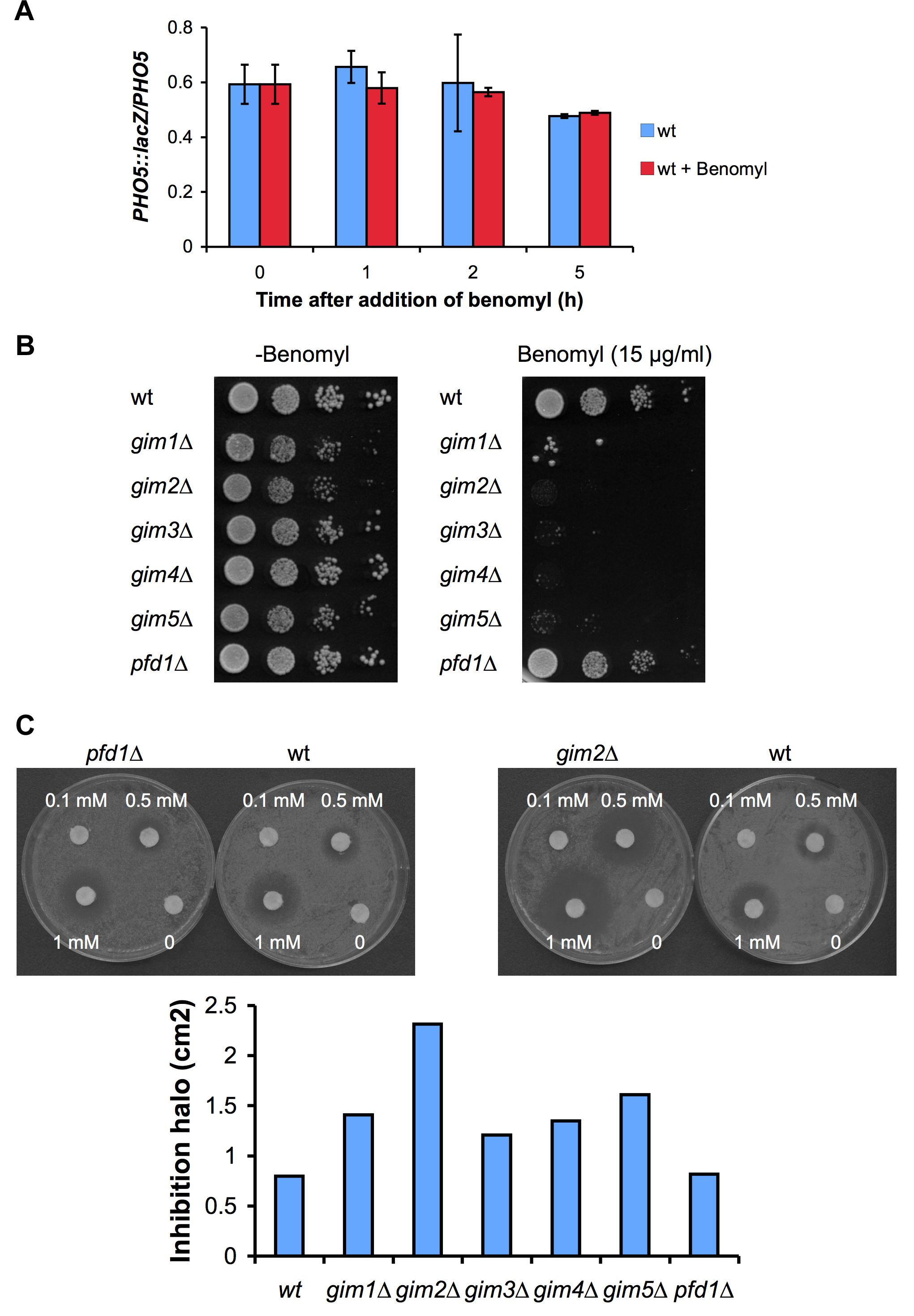

Supplement: Figure S2 — Transcriptional phenotypes of prefoldin do not correlate with sensitivity to benomyl or latrunculin A. A. GLAM ratios obtained after incubating wild-type cells in the presence of benomyl for the indicated times. 15 µg/ml benomyl was added to wild-type cells growing exponentially in selective medium. GLAM assay was performed at the indicated times. Mean and standard deviation of 3 biological replicates are shown. B. Drop assay showing that pfd1Δ exhibits much weaker sensitivity to benomyl than the other prefoldin mutants. Serial dilutions of exponentially growing cultures of the indicated strains were plated on YPD and YPD plus 15 µg/ml benomyl and incubated during 3 days at 30°C. C. Plate assay showing that gim2Δ is hypersensitive to latrunculin A, whereas pfd1Δ is not. The concentration of latrunculin A soaking each position is shown. Histograms represent the diameter of the inhibition halo of the 0.5 mM position for each strain after 2 days of incubation. (TIF) [file pgen.1003776.s002.tif]

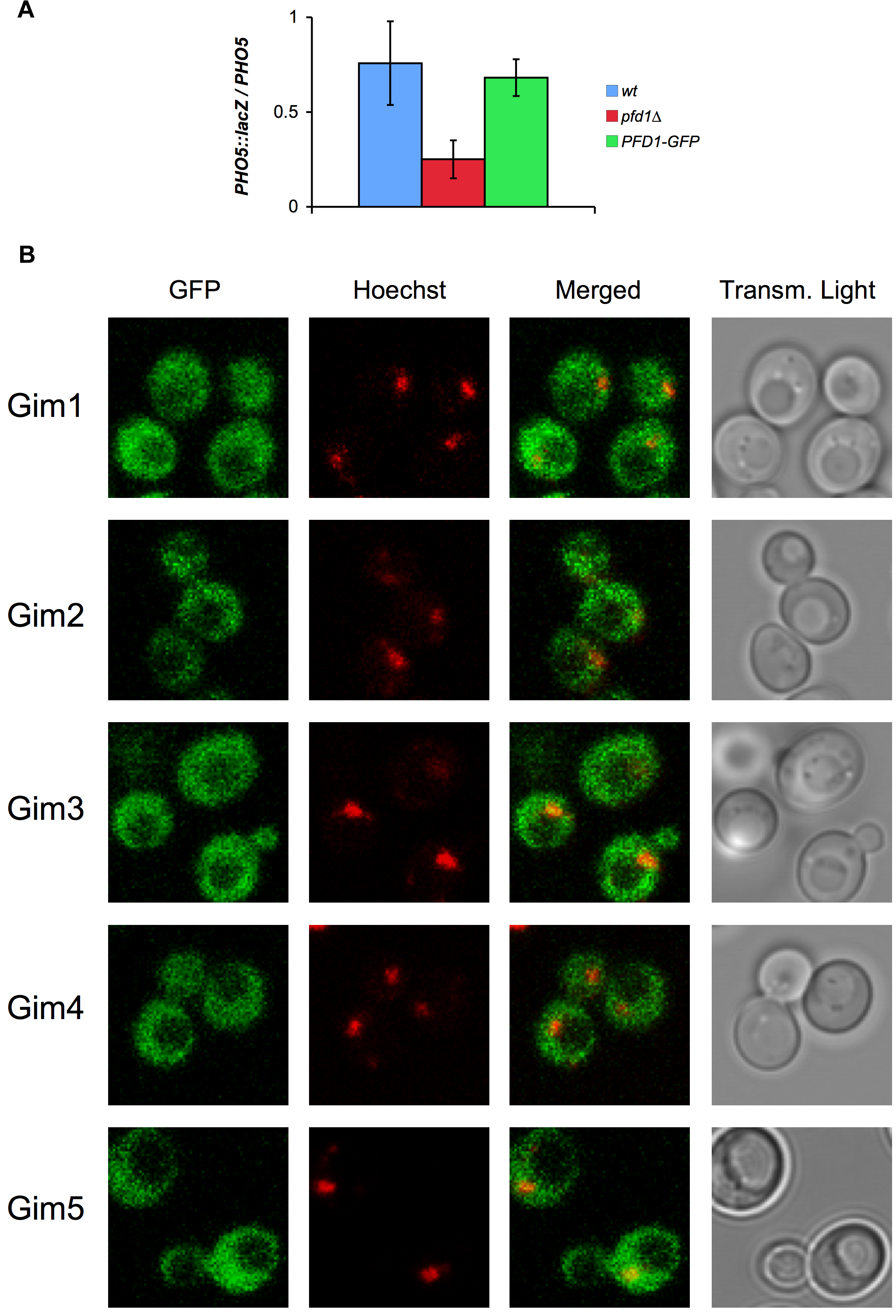

Supplement: Figure S3 — Nucleo-cytoplasmic localization of prefoldin. A. The Pfd1-GFP fusion protein is functional. GLAM assays were performed with cells from a pfd1Δ transformant expressing Pfd1-GFP, from the non-transformed mutant and from an isogenic wild-type strain. The results shown represent the mean GLAM ratios and the standard deviations of three biological replicates. B. Analysis of cells expressing the indicated GFP fusions by confocal microscopy. The results reveal nucleo-cytoplasmic distribution for these proteins. Confocal microscopy was performed as described in the Materials and methods section. Nuclei were stained with Hoechst. (TIF) [file pgen.1003776.s003.tif]

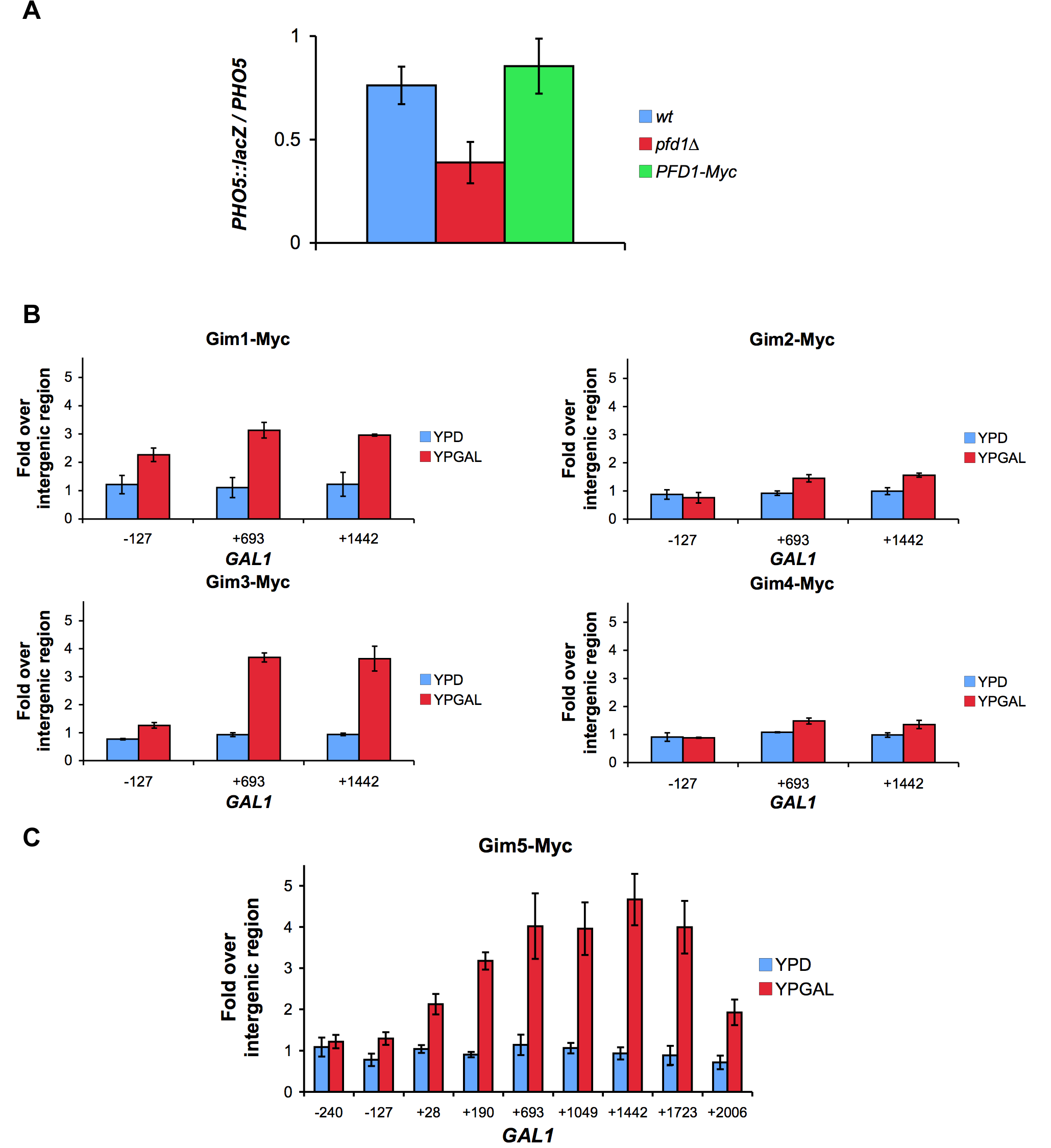

Supplement: Figure S4 — Prefoldin binds chromatin. A. The Pfd1-Myc fusion protein is functional. GLAM assays were performed with cells from a pfd1Δ transformant expressing Pfd1-Myc, from the non-transformed mutant and from an isogenic wild-type strain. The results shown represent the mean GLAM ratios and the standard deviations of three biological replicates. B. and C. Binding of Myc fusions of the indicated prefoldin subunits along GAL1 under repressed (YPD) and activating (YPGAL) conditions. Cells exponentially growing in glucose- (YPD) or in galactose-containing medium (YPGAL) were analyzed by anti-Myc ChIP, as described in the Materials and methods section, using amplicons centered in the indicated GAL1 positions. The results shown represent the means and the standard deviations of three biological replicates. (TIF) [file pgen.1003776.s004.tif]

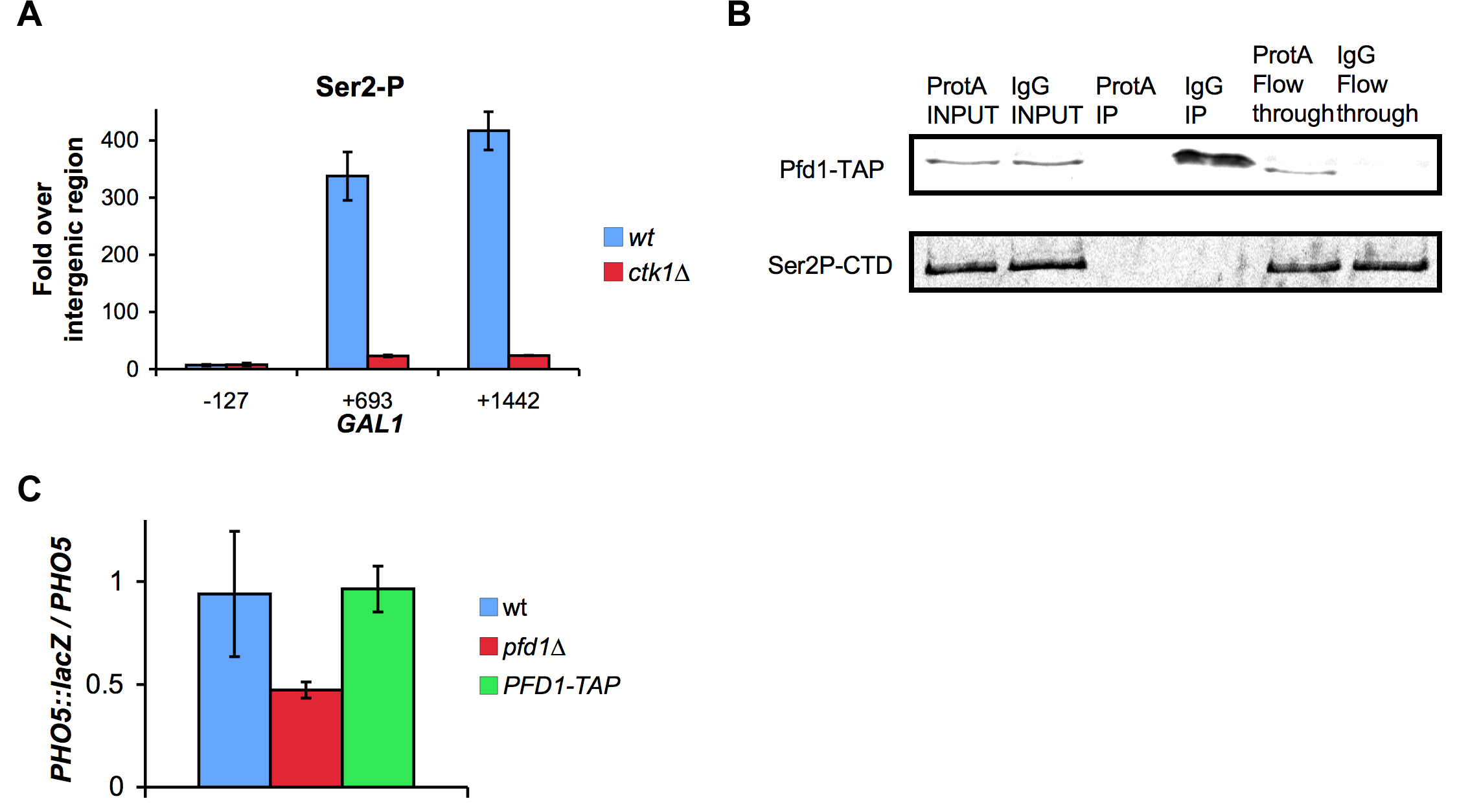

Supplement: Figure S5 — Recruitment of prefoldin to transcribed genes. A. ctk1Δ diminishes Ser2 phosphorylation of Rpb1 CTD. Occupancy of Ser2-phosphorylated RNA polymerase II was analyzed by ChIP using the same wild-type and ctk1Δ extracts of the Pfd1-Myc experiments shown in Figure 5D. The results shown represent the means and the standard deviations of three biological replicates. B. Co-immunoprecipitation experiments do not support a direct interaction of Pfd1 to Ser2-phosphorylated RNA polymerase II. 6 mg of concentrated yeast nuclear extract from Pfd1-TAP cells were incubated either with IgG-Sepharose or with Protein-A sepharose (negative control) during 4 h at 4°C on rotation. After washing extensively, half of the IP material was loaded in a 12% polyacrylamide SDS gel and analyzed for the presence of Pfd1-TAP by Western blot, and the other half was loaded in a 7% polyacrylamide SDS gel and analyzed for the presence of Ser2-phosphorylated Rpb1. In both cases 1% of the input material and 1% of the flow-through material were also analyzed. C. The Pfd1-TAP fusion protein used in the co-immunoprecipitation experiments shown above is functional. GLAM assays were performed with cells from a pfd1Δ transformant expressing Pfd1-TAP from the non-transformed mutant and from an isogenic wild-type strain. The results shown represent the mean GLAM ratios and the standard deviations of three biological replicates. (TIF) [file pgen.1003776.s005.tif]

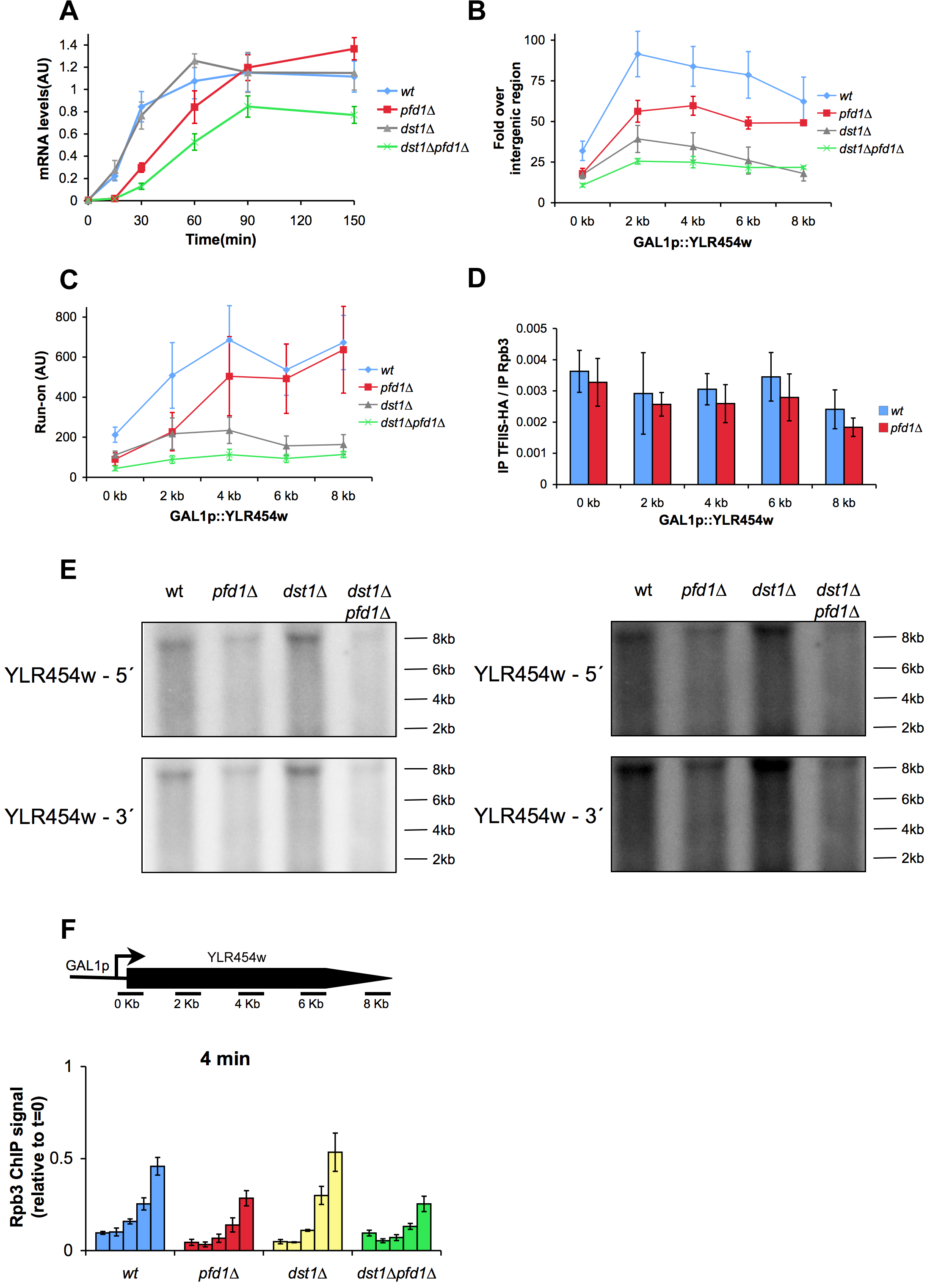

Supplement: Figure S6 — Transcriptional function of prefoldin. A. Time courses reflecting the induction of the GAL1 gene by the addition of galactose to cells grown in glycerol-lactate medium. Cells of the indicated isogenic strains were grown in glycerol-lactate medium for two generations and then 2% galactose was added to the cultures. RNA samples were then taken at the indicated times and used for Northern experiments. Mean and standard deviation of the quantitated Northern signal of three biological replicates is shown. B. RNA polymerase II distribution across GAL1p-YLR454w. Occupancy of RNA polymerase II was measured by anti-Rpb3 ChIP in the indicated isogenic strains. Points represents the amplicons described in Figure 7B. The same data as in Figure 7C are shown, except the values here are not normalized. C. Distribution of transcriptionally active RNA polymerase II across GAL1p-YLR454w, as measured by run-on. The same data of Figure 7D are shown, except values here are not normalized. D. TFIIS occupancy of GAL1p-YLR454w does not increase in the absence of prefoldin, as shown by Rpb3 and TFIIS-HA ChIP experiments. Wild-type and pfd1Δ cells expressing the HA-tagged version of TFIIS in its N-terminus and growing exponentially in YPGAL were processed for ChIP. The same extract was used to perform Anti-Rpb3 and anti-HA ChIP, in order to calculate TFIIS/Rpb3 ratios for each amplicon. Means and standard deviations of three biological replicates are shown. E. Northern blots of GAL1p-YLR454w in the indicated isogenic strains, showing the absence of the 5.3 kb cryptic transcript that these genes expressed in other genetic backgrounds [52]. The blot was hybridized with 5′ and 3′ probes in order to also explore antisense cryptic transcription. 1-day (left) and 3-days (right) exposures are shown. F. The elongation rate of RNA polymerase II in GAL1p-YLR454w is affected by the absence of Pfd1. RNA polymerase II distribution is shown, 4 min after adding 2% glucose to cells of the indicated isogenic [file pgen.1003776.s006.tif]

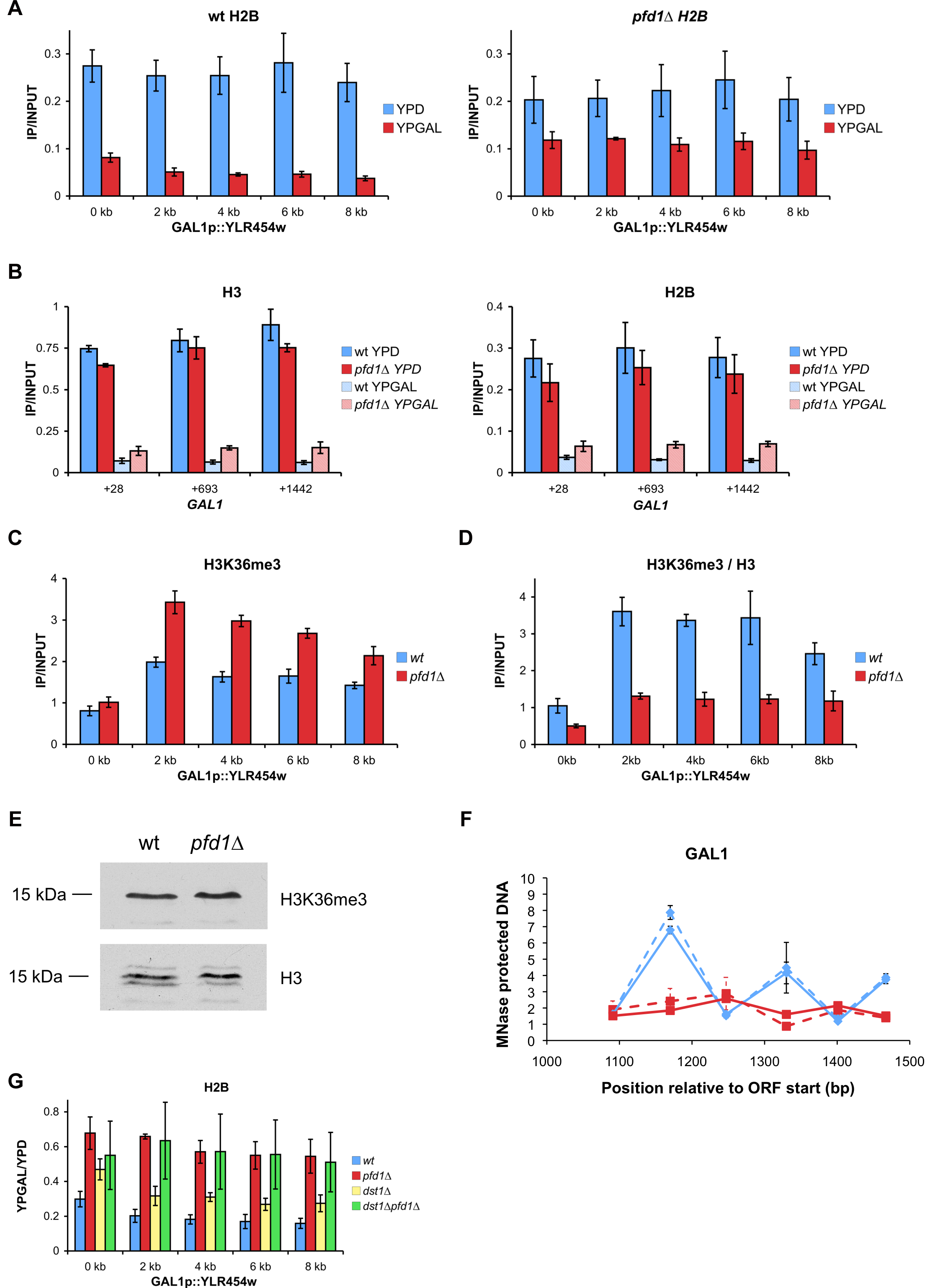

Supplement: Figure S7 — Prefoldin contribution to chromatin dynamics. A. The absence of Pfd1 impairs the characteristic difference in histone occupancy along the transcribed region that occurs between active and inactive genes. The levels of H2B bound to transcribed (YPGAL) and untranscribed (YPD) GAL1p-YLR454w were measured by ChIP in wild-type (left) and pfd1Δ (right) exponentially growing cells. B. pfd1Δ also impairs transcriptional histone dynamics in GAL1. H3 and H2B binding to GAL1 were measured in the same experiment described in A. C. and D. The chromatin of the GAL1p-YLR454w transcribed region is enriched in trimethylated H3K36, although most H3 histones accumulated in transcribed GAL1p-YLR454w in pfd1Δ are not methylated in H3K36. The levels of H3K36Me3 bound to transcribed GAL1p-YLR454w was measured by ChIP in wild-type and pfd1Δ cells exponentially growing in YPGAL (C). Levels of total H3 bound to the same gene were also measured in the same extracts and used to calculate H3K36Me3/H3 ratios. Means and standard deviation of three biological replicates are shown. E. Overall H3K36 trimethylation does not change in the absence of Pfd1. Whole cell extracts of wild-type and pfd1Δ were used to analyze the cellular levels H3K36Me3 and total H3, as described in the Materials and methods section. F. Sensitivity of GAL1 chromatin to micrococcal nuclease, under activating (YPGAL) and non-activating (YPD) conditions is not affected by the absence of Pfd1. The experiments were performed as described in Figure 8. G. Transcriptional eviction of H2B in GALp-YLR454w, expressed by the ratio between the ChIP signals of the cells grown in YPGAL and YPD, in the wild type and in the indicated isogenic mutants. (TIF) [file pgen.1003776.s007.tif]

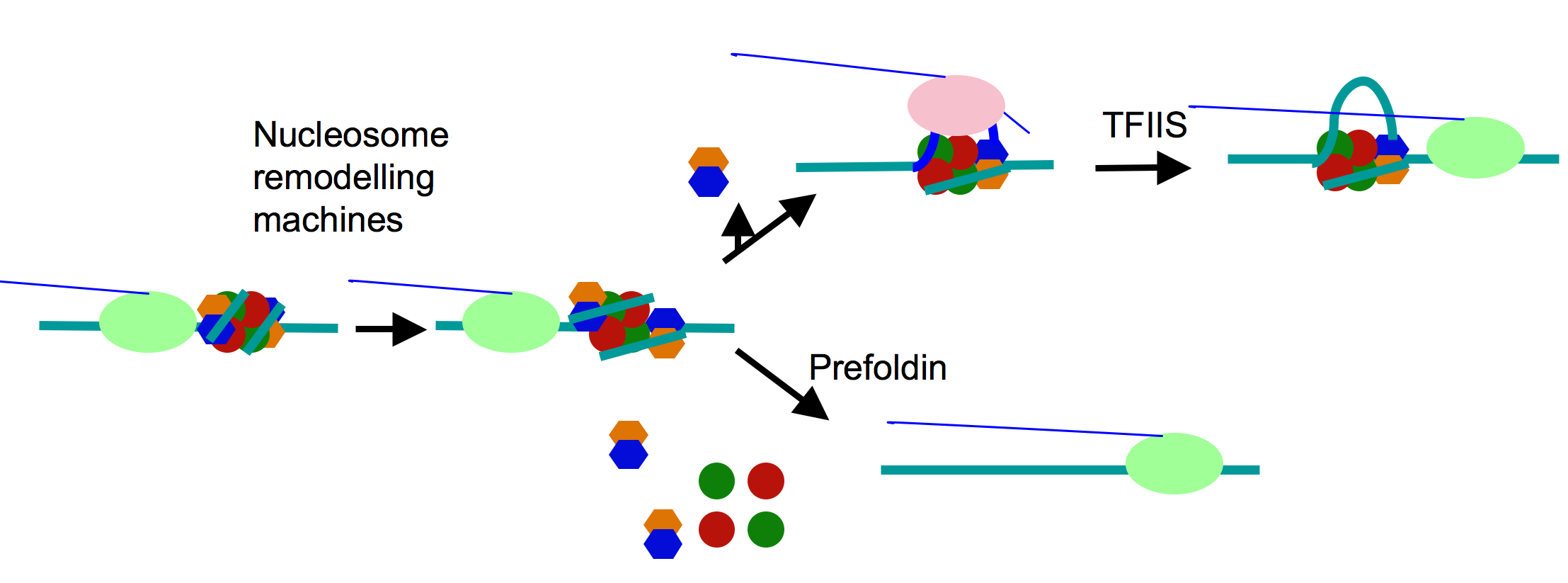

Supplement: Figure S8 — A model describing the potential role of prefoldin in histone dynamics during transcription elongation. Green ovals depict active RNA polymerase II; the pink one indicates backtracked RNA polymerase II. The other figures represent histones. (TIF) [file pgen.1003776.s008.tif]
