# Supplementary material for: The Prefoldin Complex Regulates Chromatin Dynamics during Transcription Elongation
Source: PLoS Genet. 2013 Sep 19;9(9):e1003776. doi: 10.1371/journal.pgen.1003776 (PMC3777993; doi:10.1371/journal.pgen.1003776)
Supplement: Table S2 — Strains and plasmids utilized in this work. (DOC) [file pgen.1003776.s010.doc]

| **Yeast strain** | **Relevant genotype** | **Reference** |
| --- | --- | --- |
| BY4741 | MAT a *his3∆1 leu2∆0 met15∆0 ura3∆0* | Euroscarf |
| GMY15-8b | BY4741 *pfd1::kanMX4* | This work |
| GMY10-8c | BY4741 *gim1::kanMX4* | This work |
| GMY11-8c | BY4741 *gim2::kanMX4* | This work |
| GMY12-7d | BY4741 *gim3::kanMX4* | This work |
| GMY13-5b | BY4741 *gim4::kanMX4* | This work |
| GMY14-1a | BY4741 *gim5::kanMX4* | This work |
| MMY9.2 | BY4741 *dst1::kanMX4* | Gomez-Herreros, 2012 |
| GMY38-2d | BY4741 *dst1::kanMX4* *pfd1::natMX4* | This work |
| KW610 | Mat a *ade2∆ his3∆ leu2∆ trp1∆ ura3∆ mex67::HIS3 [pUN100(CEN LEU2) mex67-5] xpo1::TRP1 xpo1-1::HIS3* | Brune, 2005 |
| Y07202 | BY4741 *trp1::kanMX4* | Euroscarf |
| Pfd1-Myc5 | BY4741 *trp1::kanMX4 PFD1·18XMyc::TRP1* | This work |
| Gim1-Myc8 | BY4741 *trp1::kanMX4 GIM1·18XMyc::TRP1* | This work |
| Gim2-Myc | BY4741 *trp1::kanMX4 GIM2·18XMyc::TRP1* | This work |
| Gim3-Myc6 | BY4741 *trp1::kanMX4 GIM3·18XMyc::TRP1* | This work |
| Gim4-Myc2 | BY4741 *trp1::kanMX4 GIM4·18XMyc::TRP1* | This work |
| Gim5-Myc | BY4741 *trp1::kanMX4 GIM5·18XMyc::TRP1* | This work |
| GMY18-8c | BY4741 *trp1::kanMX4 PFD1·18XMyc::TRP1 gim5::kanMX4* | This work |
| GMY39 | BY4741 *trp1::kanMX4 GIM5·18XMyc::TRP1 pfd1::kanMX4* | This work |
| 16.53a | BY4741 *URA3::GAL1p::YLR454w* | Malagón F. |
| GMY20-5c | BY4741 *URA3::GAL1p::YLR454w pfd1::kanMX4* | This work |
| GMY16-8d | BY4741 *URA3::GAL1p::YLR454w dst1::kanMX4* | This work |
| GMY40 | BY4741 *URA3::GAL1p::YLR454w pfd1::kanMX4 dst1::kanMX4* | This work |
| GMY19-5c | BY4741 *URA3::GAL1p::YLR454w pfd1::kanMX4 trp1::HISG DST1·3XHA::TRP1* | This work |
| XPY41 | BY4741 *trp1::kanMX4 PFD1·18XMyc::TRP1 ctk1::kanMX4* | This work |
| Pfd1-TAP | BY4741  *PFD1·TAP::HIS3* | Open Biosystems |

| **Plasmid** | **Description** | **Reference** |
| --- | --- | --- |
| YCplac33 | YCp vector based on the *URA3* gene | Morillo-Huesca, 2006 |
| pSCh202 | pRS416 containing the *PHO5* region fused to the *GAL1* promoter | Morillo-Huesca, 2006 |
| pSCh212 | pSCh202 with lacZ transcriptionally fused to the 3′-end UTR of *PHO5* | Morillo-Huesca, 2006 |
| PSCh209Lac4 | pSCh202 with K. lactis *LAC4* transcriptionally fused to the 3′-end UTR of *PHO5* | Morillo-Huesca, 2006 |
| pGMZ2 | YCplac33-yGFP with *PFD1* transcriptionally fused | This work |
| pGMZ3 | YCplac33-yGFP with *GIM1* transcriptionally fused | This work |
| pGMZ4 | YCplac33-yGFP with *GIM2* transcriptionally fused | This work |
| pGMZ5 | YCplac33-yGFP with *GIM3* transcriptionally fused | This work |
| pGMZ6 | YCplac33-yGFP with *GIM4* transcriptionally fused | This work |
| pGMZ7 | YCplac33-yGFP with *GIM5* transcriptionally fused | This work |
| pGA226 | To tag with ·18Myc::*TRP1* | Jimeno-Gonzalez |
| p448 | *PAB1*::RFP/*ADE2*/CEN | Haimovich G. |
