# Supplementary material for: The Prefoldin Complex Regulates Chromatin Dynamics during Transcription Elongation
Source: PLoS Genet. 2013 Sep 19;9(9):e1003776. doi: 10.1371/journal.pgen.1003776 (PMC3777993; doi:10.1371/journal.pgen.1003776)
Supplement: Table S3 — DNA oligonucleotides utilized in this work. (DOC) [file pgen.1003776.s011.doc]

| **Primer** | **Sequence 5’-3’** | **Use** |
| --- | --- | --- |
| 1_100.txt-3F (*YLR454w*) | GATGTTTCCGATTAATGTTCTACTGTACAA | Q-PCR |
| 1_100-66R (*YLR454w*) | GCTCCATAAGAAAGTCACTGCAAA | Q-PCR |
| 1900-2000-3F (*YLR454w*) | AGACAGAAGGAAATTTTACCAAGCG | Q-PCR |
| 1900-2000-63R (*YLR454w*) | AATCGAAAAAATCAGGTAGTTGCTG | Q-PCR |
| 3800-4100-191F (*YLR454w*) | GATATGCTTCAATCCGACAGAGAG | Q-PCR |
| 3800-4100-258R (*YLR454w*) | TCAACAGTTACCGATGGTATTAAAGG | Q-PCR |
| 5800-6100-2F (*YLR454w*) | AGCCGGACAAACAGAACAGC | Q-PCR |
| 5800-6100-71R (*YLR454w*) | CAGGGTCTTTTTGGTGTTTTTCA | Q-PCR |
| 7600_7700.txt-21F (*YLR454w*) | GTTGGACAATCTTAAAGTCGGGA | Q-PCR |
| 7600_7700.txt-92R (*YLR454w*) | GTTGGACAATCTTAAAGTCGGGA | Q-PCR |
| *GAL1*(-240)up | AAAATTGGCAGTAACCTGGCC | Q-PCR |
| *GAL1*(-240)low | CCCCAGAAATAAGGCTAAAAAACTAA | Q-PCR |
| *GAL1*(-127)up | GCGAAGCGATGATTTTTGATC | Q-PCR |
| *GAL1*(-127)low | AAGAAGTAATACAAACTGAAAATGTTGAAAG | Q-PCR |
| *GAL1*(+29)up | ACGTCAAGGAGAAAAAACTATAATGACTAA | Q-PCR |
| *GAL1*(+29)low | CGGCCAATGGTCTTGGTAATT | Q-PCR |
| *GAL1*(+190)up | TTGCTAGATCGCCTGGTAGAGTC | Q-PCR |
| *GAL1*(+190)low | GGCGCAAAGCATATCAAAATC | Q-PCR |
| *GAL1*(+693)up | GGTATGGATCAGGCTGCCTC | Q-PCR |
| *GAL1*(+693)low | AATTGCGGAAATTTAAACGGAG | Q-PCR |
| *GAL1*(+1049)up | ATCCGGCATCGAACGGTTA | Q-PCR |
| *GAL1*(+1049)low | TCAAGGATTGTGCGACATCG | Q-PCR |
| *GAL1*(+1442)up | TGCTTTGTCAAATGGATCATATGG | Q-PCR |
| *GAL1*(+1442)low | CCTGGAACCAAGTGAACAGTACAA | Q-PCR |
| *GAL1*(+1723)up | ACGAGTAGTAACACTTTTATAGTTCATACATGCT | Q-PCR |
| *GAL1*(+1723)low | AGTTTGTGGATAATCGAAATCTCTTACA | Q-PCR |
| *GAL1*(+2006)up | ATTGCCGTCTTGAAACTTTTTGTC | Q-PCR |
| *GAL1*(+2006)low | AAATCTGAATTGTCCAAATTCAGTACA | Q-PCR |
| IntergenicChrVup | tgttcctttaagaggtgatggtga | Q-PCR |
| IntergenicChrVlow | gtgcgcagtacttgtgaaaacc | Q-PCR |
| *ADH1*(-320)up | CTCCCCCGTTGTTGTCTCAC | Q-PCR |
| *ADH1*(-320)low | CAACGACATCTGTTGGTGCTGT | Q-PCR |
| *ADH1*(+210)up | GCACGGTGACTGGCCATT | Q-PCR |
| *ADH1*(+210)low | TCTTCCAGCCCTTAACGTTTTC | Q-PCR |
| *ADH1*(+833)up | ACGTCGGTAACAGAGCTGACAC | Q-PCR |
| *ADH1*(+833)low | TTCTGGCAAGGTAGACAAGCC | Q-PCR |
| *PMA1*(-404)up | AAATTGTTACTCTCACACTCTTTAGTTCGT | Q-PCR |
| *PMA1*(-404)low | GGAGCATAAGCGGTACCCAC | Q-PCR |
| *PMA1*(+198)up | GACGACGAAGACAGTGATAACGA | Q-PCR |
| *PMA1*(+198)low | GAAGTTAAACCGTAAGATGGGTCAGT | Q-PCR |
| *PMA1*(+2606)up | TGTCTGGATCTGGTCTATCGGTATC | Q-PCR |
| *PMA1*(+2606)low | GGCTTCAGAAGTGGACATTTCG | Q-PCR |
| *STL1*(-170)up | GAGAAATTGAGAAAGCTTAAGTGAGATG | Q-PCR |
| *STL1*(-170)low | TGACTACTCCTGAACTGCAATTCTG | Q-PCR |
| *STL1*(+74)up | TTTCAAAGGCAAATTTATAAGCAGAA | Q-PCR |
| *STL1*(+74)low | ACAGGGAGAAGCCCGTCATA | Q-PCR |
| *STL1*(+1385)up | TTTATTGGACAGTCCGGTTGG | Q-PCR |
| *STL1*(+1385)low | CTTCCGGCGGTTTCAGG | Q-PCR |
| *CTT1*(-190)up | TCACCCTCTGGCTGCAGG | Q-PCR |
| *CTT1*(-190)low | AAATGCACAATGTGTCCAGGC | Q-PCR |
| *CTT1*(+214)up | GAGAAAGAGTTCCGGAGCGTG | Q-PCR |
| *CTT1*(+214)low | TGGAGCGGCGTATGTAATATCAC | Q-PCR |
| *CTT1*(+1609)up | GAGTTACGCAATACTTTGGTTTGCT | Q-PCR |
| *CTT1*(+1609)low | GGCATAACCTTCAAGGTCAACAG | Q-PCR |
| *GRE2*(-290)up | ATTAATATCAATGCTGCAATACCTTCTC | Q-PCR |
| *GRE2*(-290)low | TTATATGCTTCTTACTTATTTTATCAATGGC | Q-PCR |
| *GRE2*(+175)up | AGGCCTTTGGTAACAACCCA | Q-PCR |
| *GRE2*(+175)low | GATATCCTTGCCGTGCTTTTG | Q-PCR |
| *GRE2*(+948)up | ACCCATAACACCCTTGGTGC | Q-PCR |
| *GRE2*(+948)low | GAGGCAGTGTCGTCAATGGTC | Q-PCR |
| YLR454W-103R | TATTTATTCCTAAAAGCTTC | Q-PCR |
| YLR454W-88F | CTTTTAGGAATAAATATAAC | Q-PCR |
| YLR454W-180R | AACGGATTTTAGCCTGACGG | Q-PCR |
| YLR454W-161F | CCGTCAGGCTAAAATCCGTTCG | Q-PCR |
| YLR454W-250R | TAGTAGATGATTTCTTGGAATC | Q-PCR |
| YLR454W-232F | TCCAAGAAATCATCTACTAATG | Q-PCR |
| YLR454W-331R | AAATGAAGCCCCCACCATTATC | Q-PCR |
| YLR454W-311F | ATAATGGTGGGGGCTTCATTTC | Q-PCR |
| YLR454W-409R | CGTTGTTGACTAATTGCGTATC | Q-PCR |
| YLR454W-388F | GATACGCAATTAGTCAACAACG | Q-PCR |
| YLR454W-491R | AAAAAGGAATCAAATCTTAGTG | Q-PCR |
| YLR454W-7162F | GTAAAAATGATGCAAATATTTG | Q-PCR |
| YLR454W-7259R | AAACCGACTGTGTCATTACCAC | Q-PCR |
| YLR454W-7240F | GGTAATGACACAGTCGGTTTGG | Q-PCR |
| YLR454W-7337R | ACGTTTTTAACAACCTTAATAC | Q-PCR |
| YLR454W-7321F | AAGGTTGTTAAAAACGTTGAGAC | Q-PCR |
| YLR454W-7422R | AACTCCTTTAACGATAACCACTC | Q-PCR |
| YLR454W-7406F | TATCGTTAAAGGAGTTAATTTAC | Q-PCR |
| YLR454W-7499R | CCTTCCTCTGATTCTGATGACAC | Q-PCR |
| YLR454W-7477F | GTGTCATCAGAATCAGAGGAAGG | Q-PCR |
| YLR454W-7581R | ATCTTCAACGATCATATAATCAC | Q-PCR |
| YLR454W-7561F | GATTATATGATCGTTGAAGATC | Q-PCR |
| GAL1-1040F | AAGAGTCTCTCGCCAATAAGAAAC | Q-PCR |
| GAL1-1142R | GATGTTGTTAAGTAGTCTCTTGTG | Q-PCR |
| GAL1-1119F | CACAAGAGACTACTTAACAACATC | Q-PCR |
| GAL1-1221R | AGCCTTCAAGACTCTTAAAGATTC | Q-PCR |
| GAL1-1199F | AATCTTTAAGAGTCTTGAAGGCTG | Q-PCR |
| GAL1-1296R | CTCGTTCATCAAGGCACCAAATTG | Q-PCR |
| GAL1-1273F | CAATTTGGTGCCTTGATGAACGAG | Q-PCR |
| GAL1-1386R | ACCATATGATCCATTTGACAAAGC | Q-PCR |
| GAL1-1415F | GTTGTACTGTTCACTTGGTTCCAG | Q-PCR |
| GAL1-1520R | TCAGTGATCTTAGGGTACTTGACC | Q-PCR |
| CDC10-5-383 | AAGAATTGACAGCCCAACGTG | Q-PCR |
| CDC10-5-478 | GGCGGCTCAACTCCTTTCC | Q-PCR |
| YLR(0kb)up | ACAAGTGGCTTATATTTGCAGTGACT | Run-on Probe |
| YLR(0kb)low | ATGAAGCCCCCACCATTATCT | Run-on Probe |
| YLR(2kb)up | CGCTGATATCGACACGCTAATG | Run-on Probe |
| YLR(2kb)low | CATAAAAACCGACCTAGCACCTAG | Run-on Probe |
| YLR(4kb)up | CAAACTTCATTCACGATGTTGGAA | Run-on Probe |
| YLR(4kb)low | GGCACGAACAACGAGTAATATCTATCT | Run-on Probe |
| YLR(6kb)up | ACAGCCTTTCCATTTCGACTCTT | Run-on Probe |
| YLR(6kb)low | TCATCGCAGCTATGACATTGTTTA | Run-on Probe |
| YLR(8kb)up | TTCAATGAAATGTTAAAGAGATCAAGTG | Run-on Probe |
| YLR(8kb)low | GTGGTGAAGTGTCGTCAGCAAT | Run-on Probe |
| PFD1GFPup | GAATTCTTTGCCTTCAAGTGTTTTGT | Cloning |
| PFD1GFPlow | CTGCAGATTCTTCATCAATGCCTTTA | Cloning |
| Gim1GFPup: | gaattcACAAGCATCGCTATAAATGA | Cloning |
| Gim1GFPlow: | ctgcagCCTTCCTGGGCCAGTGGA | Cloning |
| GIM2GFPup: | GAATTCTGATTTGTTTCGTTTCTTCG | Cloning |
| GIM2GFPlow: | GCATGCTATCTTGAGGTTTTTAGTCC | Cloning |
| Gim3GFPup: | ggatccCAGTGACTCAACGAGTTAAC | Cloning |
| Gim3GFPlow: | ctgcagACGTTCAAGGTTGATATTAT | Cloning |
| Gim4GFPup | ggatccATGAACACACATTATAAACT | Cloning |
| Gim4GFPlow: | ctgcagGTTTTTAACGACTTGAATCT | Cloning |
| GIM5GFPup: | GAATTCTTTTTTGTGTTGCGTGGCTG | Cloning |
| GIM5GFPlow: | GCATGCGGCTGTAGACGACTCCTTTT | Cloning |
| PFD1Myc-up: | actgttgaaaaaacaatagacaatctaaaggcattgatgaagaatTCCGGTTCTGCTGCTAG | Cloning |
| PFD1Myc-low: | agtaagttttccggctaagaaaggaaaggctattgccgctttcttCCTCGAGGCCAGAAGAC | Cloning |
| GIM1Myc-up: | CTAATTAAACTGAACAATACAGCAGCTTCCACTGGCCCAGGAAGGTCCGGTTCTGCTGCTAG | Cloning |
| GIM1Myc-low: | TAAGTTACATAATTGTATATAAAAGAAAATTTTATTTTGGGCAGACCTCGAGGCCAGAAGAC | Cloning |
| GIM2Myc-up: | CAAGACTTGAAGCAGGCTCAAGAAGGGACTAAAAACCTCAAGATATCCGGTTCTGCTGCTAG | Cloning |
| GIM2Myc-low: | AGGTTGATTGTGTTGTTCTATGTAGTGCCCTTTATTTTTCTTTGGCCTCGAGGCCAGAAGAC | Cloning |
| GIM3Myc-up: | CTAATTAAACTGAACAATACAGCAGCTTCCACTGGCCCAGGAAGGTCCGGTTCTGCTGCTAG | Cloning |
| GIM3Myc-low: | TCTTTACTTTTCTTTAATTTCCCTCCTGAAAAAATTAAAAATTTTCCTCGAGGCCAGAAGAC | Cloning |
| GIM4Myc-up: | TTTGAAAAATGGAAAAAAGACAATAAGATTCAAGTCGTTAAAAACTCCGGTTCTGCTGCTAG | Cloning |
| GIM4Myc-low: | TAAATGGCTCACATAAATACATAAGAGTACCTGATTGTGTTTATTCCTCGAGGCCAGAAGAC | Cloning |
| GIM5Myc-low: | AGTAAACAACAACAGCAGCAACAAAAAAAGGAGTCGTCTACAGCCTCCGGTTCTGCTGCTAG | Cloning |
| GIM5Myc-low: | TTGCATATAGATAAACGTATATCAGATGGCTACAAATGAGATATGCCTCGAGGCCAGAAGAC | Cloning |
